# The presence of Superfund sites as a determinant of life expectancy in the United States

**DOI:** 10.1038/s41467-021-22249-2

**Published:** 2021-04-13

**Authors:** Amin Kiaghadi, Hanadi S. Rifai, Clint N. Dawson

**Affiliations:** 1 Civil and Environmental Engineering, University of Houston, Houston, TX USA; 2 Oden Institute for Computational Engineering and Sciences, University of Texas at Austin, Austin, TX USA

**Keywords:** Environmental sciences, Environmental impact, Natural hazards

## Abstract

Superfund sites could affect life expectancy (LE) via increasing the likelihood of exposure to toxic chemicals. Here, we assess to what extent such presence could alter the LE independently and in the context of sociodemographic determinants. A nationwide geocoded statistical modeling at the census tract level was undertaken to estimate the magnitude of impact. Results showed a significant difference in LE among census tracts with at least one Superfund site and their neighboring tracts with no sites. The presence of a Superfund site could cause a decrease of −0.186 ± 0.027 years in LE. This adverse effect could be as high as −1.22 years in tracts with Superfund sites and high sociodemographic disadvantage. Specific characteristics of Superfund sites such as being prone to flooding and the absence of a cleanup strategy could amplify the adverse effect. Furthermore, the presence of Superfund sites amplifies the negative influence of sociodemographic factors at lower LEs.

## Abstract (web summary)

Superfund sites have hazardous wastes that could affect the health of those who live near them, but this has not been assessed across the USA. Here the authors find that proximity to superfund sites decreases life expectancy and is further exacerbated by sociodemographic and climate change factors.

## Introduction

Life expectancy (LE) is one of the most basic yet important indicators of public health^1,2^. Studies showed a 1% increase in LE could lead to a 1.7–2% increase in population^3^. The observed discrepancy in LE around the globe is a direct result of inequalities in mortality risks^4^. The latter has been associated, by many researchers, with sociodemographic variables (e.g. race/ethnicity, sex, income, age, sanitation, and education), as well as, the spread of different communicable and non-communicable diseases (NCDs) such as diarrhea, HIV, and cancer^5–7^. In developed countries, such as in the U.S., where the majority of the population has access to basic health services^8^, the cause of specific NCDs could be attributed to exposure to chemical and biological hazards from various sources^9–12^.

While many studies have broken down the mortality rates associated with different diseases, only a few have paid attention to hazardous waste and Superfund sites and their potential impact on mortality rates. The presence of these sites could be considered a contributing factor affecting LEs through releases of hazardous/toxic contaminants^13–15^ and potential acute and chronic exposure to the pollutants contained within them^16^. For most Superfund sites, cleanup actions did not start till the 1980s, even though their presence dates as far back as the 1930s and 1940s. Considering the fact that the average LE in the U.S. is 78.7 years^17^ and millions of children have been raised within less than a 1.61 km (1-mile) radius from a federally designated Superfund site^18^, it is necessary to understand to what extent the presence of Superfund sites could affect LE.

Furthermore, and when taken in the context of natural disasters and climate change, it becomes even more critical to understand the association between hazardous waste and Superfund sites, human health, and LE. The literature provides ample evidence that contaminant releases from anthropogenic sources (e.g., petrochemicals or hazardous waste sites) could increase the mortality rate in fence-line communities^10,19–22^. However, inconsistent results were also reported; one study showed no overall maternal-fetal death associated with residential proximity to hazardous waste sites^23^ while another study showed an increased risk of congenital anomalies due to proximity to Superfund sites that had not been remediated^24^. Moreover, at least one study in the 28 member states of the European Union revealed a significant positive correlation between exposure to benzene emissions and mortality rates among people who live in the vicinity of emission sources^19^. Other recent studies in the US also showed a significant correlation between the residential proximity to Superfund sites and the occurrence of non-Hodgkin’s lymphoma, especially among males^10,25^.

While some studies questioned the essence and value of cleanup actions at Superfund sites based on their effect on housing market outcomes^26^, it has been shown that Superfund sites (at least the ones with completed human exposure pathways) without any remediation strategy could cause billions of dollars in the form of medical costs and lost productivity alone^20^. Studies have also argued that constant exposure of fence-line communities to hazardous contaminants before, during, and even after cleanup activities could cause a long-lasting effect on public health and ecosystems^27–29^. It is important to note that almost none of the aforementioned studies provide a comprehensive analysis at the national level on the impact of Superfund sites on LE.

This study provides an overall estimation of the impact of living near a Superfund site on general health (using LE as a surrogate) at the national level by considering Superfund sites as a single source of exposure regardless of their contaminants of concern. Moreover, given the recent report by the Government Accountability Office (GAO) that revealed that approximately 60% of Superfund sites managed by EPA could potentially be affected by natural hazards (e.g., flooding and wildfire)^30^, this study explores the associations between the flooding potential at Superfund sites and its role in LE. Flooding, in addition to inundation of affected land areas, could facilitate the transport of contaminants from Superfund sites and potentially affect neighborhoods farther than the nearby fence-line communities;^14,31,32^ such effects can potentially be exacerbated by a changing future climate^30,33^. Thus, it is essential to understand to what extent being located in a Federal Emergency Management Agency (FEMA) defined floodplain could influence the effect of Superfund sites on LE.

The study presents a nationwide geocoded statistical modeling analysis of the presence of Superfund sites, their flood potential, and the impact on LE independently and in the context of other sociodemographic determinants. There is no comprehensive study at the national level with a geographic scale smaller than the county-level on the effect of Superfund sites on LE. The present study analyzes the aforementioned potential correlations at the census tract level. Furthermore, and unlike prior studies that have focused mainly on sites on the National Priorities List (NPL, ~1300 sites)^34^, this study includes sites proposed to be on the list, removed from the list, waiting to become part of the list and sites that are not overseen by the EPA (an additional ~11,700 sites)^35^.

The study more specifically undertakes statistical modeling to answer the following questions: (1) Does the presence of a Superfund site within a census tract independently cause a significant change in LE when compared to its immediate neighboring tracts that do not have a Superfund site (the qualification question)? (2) What is the magnitude and extent of change in LE that this presence could cause (the quantification question) when sociodemographic determinants are also considered? (3) How does the effect of Superfund sites on LE vary in tracts with different sociodemographic characteristics?; and (4) How does the vulnerability to flooding of Superfund sites and their cleanup status amplify or reduce the magnitude of the effect on LE?

## Results and discussion

A summary of statistics for LE and all sociodemographic variables in all 65,226-census tracts is provided in Supplementary Table 1 in the Supplementary Information (SI). The median LE values for all census tracts, tracts with at least one Superfund site, and tracts with no sites were found to be 78.50, 77.50, and 78.7 years, respectively, indicating a difference of −1 and −1.2 years in LE between census tracts with sites when compared to overall and no sites. Such difference, however, could be a combined effect of the sociodemographic variables and Superfund sites together as will be discussed in more detail in the following sections. The results of Spearman’s rank correlation analysis are shown in Supplementary Table 2 in the SI. All of the selected sociodemographic variables showed a significant (*P*-value < 0.01) correlation with LE. However, some of the sociodemographic variables showed significant and very strong pairwise correlations with each other; for instance, median income, income per capita, and percent of the population below the poverty line (*P*-value ≪ 0.01, *R* > 0.85). For such cases, only one variable was selected for further analyses and statistical regression modeling; these were LE, the presence of Superfund sites, percent of the population above 60 years old (variable name: above 60), median income in U.S. dollars (variable name: income), percent white (variable name: white), percent of the population with at least one disability (variable name: disability), percent married people (variable name: married), percent of the population with at least one health insurance plan (variable name: insurance), percent of the population with education beyond high school diploma (variable name: education), and percent of U.S. citizens in each tract (variable name: citizenship). Supplementary Figs. 1–8 in the SI, show the distribution of these variables in all of the census tracts, with available data, in the US.

### The presence of Superfund sites and their impact on LE

Table 1 shows the *P*-values for the Mann–Whitney U test conducted for matching analyses. The results showed a significant difference (*P*-value < 0.05) in LE among tracts with at least one Superfund site and tracts with no sites. As shown in Table 1, the difference was not significant in general when all tracts, regardless of their Superfund site status, were compared to their neighbors. The *t*-test for LE also showed a significant difference among tracts with Superfund sites and their neighbors (*P*-value = 8.96E−15) and non-significant in general (*P*-value = 0.06). This result suggests the LE could be different in two neighboring tracts because of the presence of Superfund sites (due, for example, to a higher chance of exposure to a specific toxic chemical as discussed in the literature^20,36^).

**Table 1:** *P*-values for the Mann–Whitney U test (two-sided) conducted in matching analyses.

Variable	Mann–Whitney U test	
	Tracts with Superfund sites vs. neighbor tracts	All tracts vs. neighbor tracts
LE	**1.37E−13**	7.48E−01
Above60	**1.06E−04**	9.58E−01
White	**1.78E−04**	7.22E−01
Income	**0.00E**+**00**	**5.92E−07**
Insurance	**6.96E−07**	**2.97E−03**
Married	**0.00E**+**00**	**3.68E−07**
Education	**7.76E−10**	3.92E−01
Citizenship	7.02E−01	5.30E−01
Disability	**8.74E−03**	**1.29E−04**

Bold values indicate significant differences at the 0.05 confidence level.

The Mann–Whitney U test also showed significant differences for all sociodemographic variables except citizenship among tracts with Superfund sites and the median of their surrounding neighbors (confounding effect). There were no racial and educational differences in general, while significant differences were observed when only tracts with Superfund sites were compared with their neighbors. For income, marital status, and insurance coverage, both group tests showed significant differences, and for citizenship, both experiments showed no significant differences. The fact that sociodemographic variables also showed a significant difference could make the conclusion mentioned above less related to the presence of the sites exclusively. However, it would justify the inclusion of the sociodemographic variables within the ordinary least squares (OLS) model to offset the confounding effect. Furthermore, and from these observations, one could conclude that less-educated minorities (the direction of difference was determined by looking at the medians) are living in tracts with at least one Superfund site. In other words, the population living in the vicinity of Superfund sites has already been made more vulnerable to exposure to different contaminants emanating from the sites due to the higher level of social and health-related disadvantages.

### Regression analyses and Random Forests modeling (quantification)

Supplementary Table 4 in the SI shows the coefficients and model metrics (performance) for all of the nine developed OLS regression models (manual stepwise). As shown in the table, the Superfund site coefficient in the OLS model, developed solely based on the Superfund site, was significant with a relatively large coefficient (−1.146). However, considering the confounding effect of the sociodemographic variables (especially income and race), adding them to the model made the Superfund coefficient smaller (−0.186 in the final model), yet significant, and the model performance better (*R*^2^ from 0.013 to 0.546). The difference between the unadjusted analysis and adjusted for 8 potential confounders analysis may suggest that the residual confounding could account for the remaining association detected in multivariable models. In other words, there might be other variables (not considered in this study) that could adjust the effect of Superfund Sites on LE, and considering them may lower the lost years of LE associated with living in a census track with a hazardous site. Future works could include identifying such confounders and investigate their effects on the effect of Superfund sites on LE. The final developed OLS regression model, including all sociodemographic variables, was:

1 $${\mathrm{LE}} =	 \, 74.409 + 0.053\,\left( {{\mathrm{Above}}\,60} \right) + 0.026\,\left( {{\mathrm{White}}} \right)\\ \,	+ \,0.236\,\left( {{\mathrm{Income}}} \right) + 0.031\,\left( {{\mathrm{Insurance}}} \right)\\ \,	+ \,0.063\,\left( {{\mathrm{Married}}} \right) + 0.068\,\left( {{\mathrm{Education}}} \right) - 0.11\,\left( {{\mathrm{Citizenship}}} \right)\\ \,	- 0.013\,\left( {{\mathrm{Disability}}} \right) - 0.186\,\left( {{\mathrm{Superfund}}\;{\mathrm{site}}} \right)$$

**Fig. 1:**
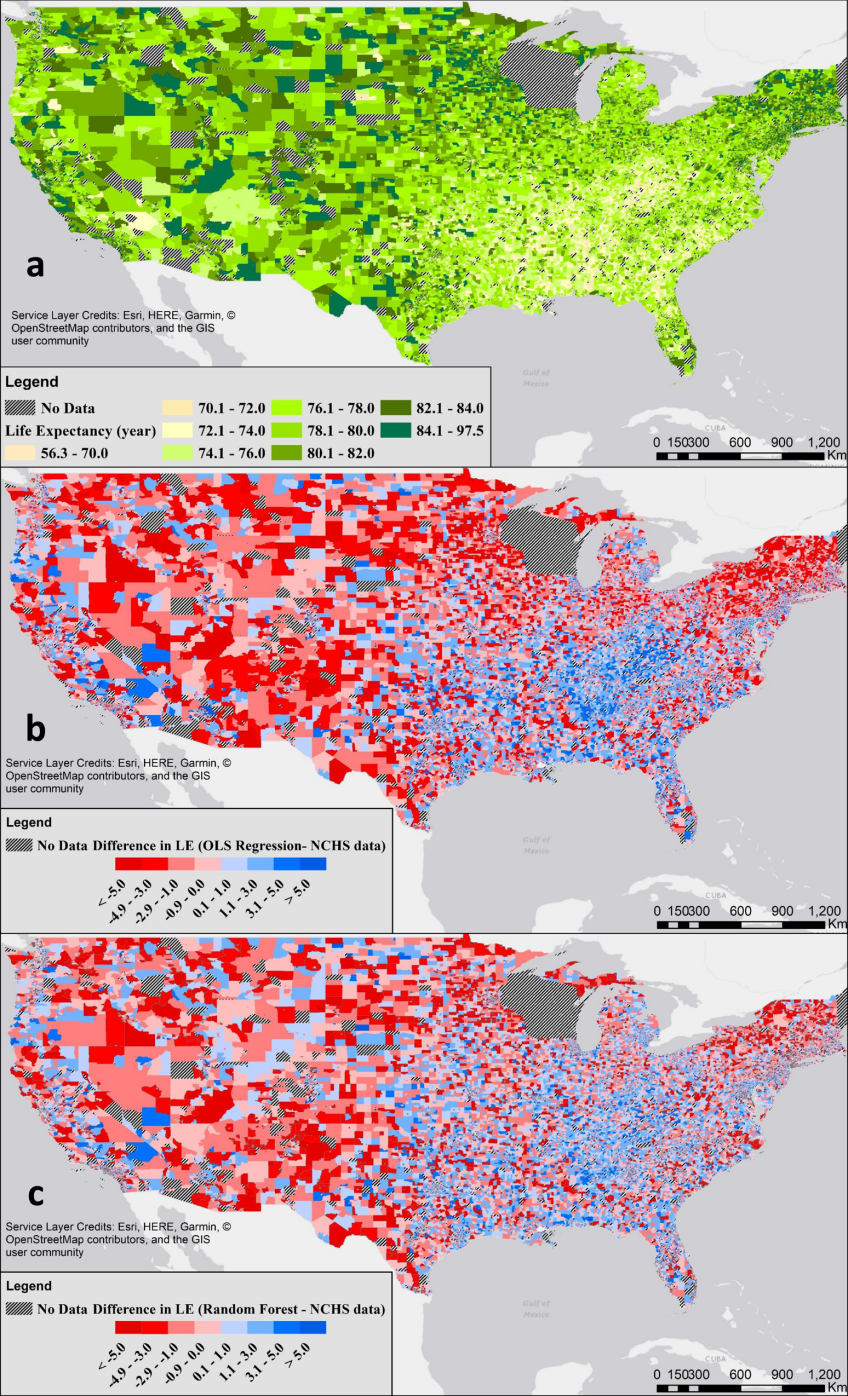
Differences between modeled and estimated Life expectancies (LEs) across the US. **a** LE in the US estimated by the National Center for Health Statistics (NCHS). **b** Difference between LE predicted by the ordinary least squares (OLS) regression model and NCHS data. **c** Difference between LE predicted by the Random Forests model and NCHS data. Out of the 72,268 census tracts within the contiguous U.S., LE data were available for 65,226 tracts.

The coefficient of determination (*R*^2^) was 0.546 for the developed OLS model and *P*-value was smaller than 0.0001 for all of the coefficients. Table 2 shows the statistics for the developed model and its coefficients. The distribution of LE in all census tracts with provided information from the NCHS is shown in Fig. 1a. The difference between the estimated LE using the OLS regression model and the NCHS database is shown in Fig. 1b.

**Table 2:** General statistics of the ordinary least squares (OLS) regression model and reported coefficients for all included variables.

Model statistics					
*R*	*R*^2^	Adjusted *R*^2^	RMSE	df	*F*
0.739	0.546	0.546	2.694	9	8709.03
Coefficient statistics					
Variable	unstandardized coefficients	Std. error	Standardized coefficients	*t*	Sig.
(Constant)	74.409	0.128		583.1	0.0E+00
Above60	0.053	0.002	0.110	33.3	1.5E-241
White	0.026	0.001	0.164	46.3	0.0E+00
Income	0.236	0.007	0.172	36.0	1.4E-281
Insurance	0.031	0.002	0.067	18.3	5.5E-75
Married	0.063	0.001	0.193	47.5	0.0E+00
Education	0.068	0.001	0.295	70.8	0.0E+00
Citizenship	−0.110	0.001	−0.376	−125.2	0.0E+00
Disability	−0.013	0.003	−0.011	−4.0	7.3E-05
Superfund site	−0.186	0.027	−0.018	−6.9	4.4E-12

*R*: correlation between the NCHS and predicted LEs, *R*^2^: coefficient of determination, Adjusted *R*^2^: adjusted *R*^2^ based on the number of predictors, *RMSE* root mean square error, *df* degrees of freedom, *F* mean square regression divided by the mean square residual, *Sig. P*-value.

Figure 1c shows the ML algorithm (RF) error in calculating LE compared to the NCHS database for all census tracts, with available data, in the U.S. By comparing Fig. 1b, c, it can be seen that the performance of the RF model was slightly better compared to the OLS regression model. This better performance was also observed in the RMSE and coefficient of determination; for the validation dataset (chosen randomly, *N* = 15,656), the RMSE was 2.578 and 2.908 years for the RFs and OLS model, respectively. The ML model, which is a complex non-linear model, could explain 0.33 years more of the variability in the LE validation data compared to the OLS model. However, the final OLS regression model had an RMSE of 2.694 years using all data. The RF model also showed slightly superior performance with regards to the slope of the trend line when the observed LEs were plotted against the modeled ones (see Supplementary Fig. 9). From the evaluation metrics, it could be concluded that the ML model showed relatively better performance; however, the similar values among all evaluation metrics justify the use of the OLS model.

Even by using a complex non-linear model, there are still 2.578 years that could not be explained. Adding more sociodemographic variables such as percent Hispanic, percent African–American, and population below the poverty line had an impact of less than 1% on the RMSE. The relatively high RMSE could be a result of other variables not considered in the models (residual confounding) such as stress level, for instance. Although the unexplained RMSE is relatively high (and may be perceived as limiting this work), in the context of the effect of the selected variables; the high adjusted *R*^2^ (0.546), the significance (*P*-Value ≪ 0.01) of all coefficients, and the fact that there exists a causal effect relationship among the selected variables and LE both point to the reliability of results from the developed regression model. Furthermore, the effect of the Superfund site becomes more influential (up to −1.223 years) among certain stratifications of sociodemographic variables, compared to its average effect (−0.186 years) as discussed below. There are likely other variables that could affect LE, and considering them may lower the RMSE, however, such investigation is beyond the scope of this work and can be addressed in future work. It should be noted that none of the studies with a similar approach have reported the RMSE value for their models and only reported the adjusted *R*^2^. The achieved *R*^2^ from the OLS regression model developed in this study is higher than most of the other studies that had values that ranged from 0.1^10^ to 0.65^19^.

Among the input variables to the OLS model, Above60, white, income, insurance, married, and education have positive coefficients, while the rest have negative coefficients indicating negative effects on LE. Income and Superfund site had the highest and lowest impacts, respectively. As noted before, sociodemographic variables (except income) were input to the model as a percent between 0% and 100%, income in multiples of $10,000, and Superfund site as a binary (0 and 1) variable. Thus, from the results of the regression analysis, it could be concluded that a 1% increase in the percent of white persons could lead to an increase of 0.026 ± 0.001 years to the LE while an increase of $10,000 in median income increases the LE by 0.236 ± 0.007 years. For the presence of a Superfund site, this number is −0.186 ± 0.027, indicating a decrease in the LE. In order to place these findings in context (i.e., the effect of Superfund site on LE), they are compared to values from other studies. Bennett et al. (2019)^12^ reported an increase of 0.61 ± 0.20 years per decrease of 10 μg/m^3^ in fine particulate matter concentration in air. Smoking could reduce the LE by 1–10 years, depending on the location, sex, and amount of use^37–39^. Reducing excessive sitting to less than three hours a day and watching TV to less than two hours a day could increase LE by 2.04 ± 0.65 and 1.495 ± 1.015 years, respectively^40^. Finally, Baars et al. ^6^ showed that the consumption of fruit and vegetables could reduce the inequalities in disability-free LE between 0.1 and 1.8 years.

### Most vulnerable population and Superfund characteristics

**Fig. 2:**
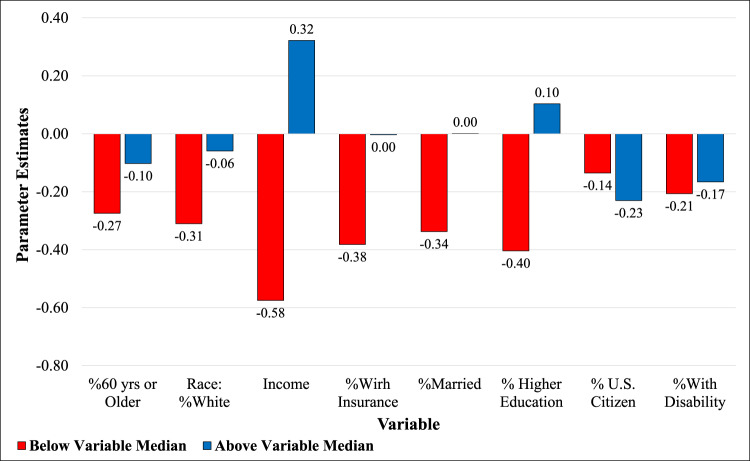
Effect modification. The effect of Superfund presence on life expectancy (LE) in tracts with at least one Superfund site with sociodemographic variables below and above the national median values. The effect modification was done by conducting separate ordinary least squares (OLS) regression analysis with a dummy variable. The effect modification analysis revealed that out of 12,717 census tracts with at least one Superfund site.

The effect modification analysis revealed that out of 12,717 census tracts with at least one Superfund site, the adverse effect of this presence was more severe on the ones with higher sociodemographic disadvantage. Figure 2 shows the effect of Superfund presence on LE (β_Superfund Site_) in tracts with sociodemographic variables below and above the national median values. This figure is a result of running a series of models explained in “Methods” section within the tracts with at least one Superfund site. For instance, when considering income, Fig. 2 shows the effect of Superfund Sites on LE within tracts with at least one site for population with an income below and above the national median. The presence of a Superfund site in a census tract with smaller than median income ($52,580) could reduce the LE by as much as 0.58 years (−0.58 as the β_Superfund Site_ when β _binary dummy for income_ is zero). This reduction could go as high as 1.223 years for tracts in the lower 10% percentile income. Interestingly, high income could completely offset the harmful effect of Superfund sites as shown in Fig. 2. For tracts with income higher than the national median, the effect of Superfund Site on LE was calculated by summing the β_Superfund Site_ and β _binary dummy for income_ (−0.58 + 0.90 = 0.32 years, where 0.90 years represent the combined effect of Superfund site and higher than median income). This improving impact could be explained by the fact that wealthier people living in a tract with a Superfund site have access to better homes (e.g., farther away from exposure paths^41^) and health care systems resulting in less exposure and better health conditions. Similar patterns were observed for education, race, being married, and health insurance coverage. Such an increase in the severity of the harmful effects in tracts with more sociodemographic disadvantage is consistent with the findings from other studies^5,22,42–46^. Disability did not show a noteworthy difference (0.04 years) between below and above the median values (even for the low 10% percentile). For Above60, tracts with higher rates of seniors showed lower harmful effects of Superfund sites compared to tracts with the younger population. This outcome could be because higher rates of Above60 directly affect the LE (make it longer) and the fact that in regions with the harmful effect of Superfund sites, the premature death rate could increase. An increase in citizenship showed a higher adverse effect of Superfund sites on LE compared to tracks with lower citizenship rates. In general, lower citizenship means higher immigrants, which potentially could mean lower residential time within a neighborhood and eventually shorter exposure time.

**Fig. 3:**
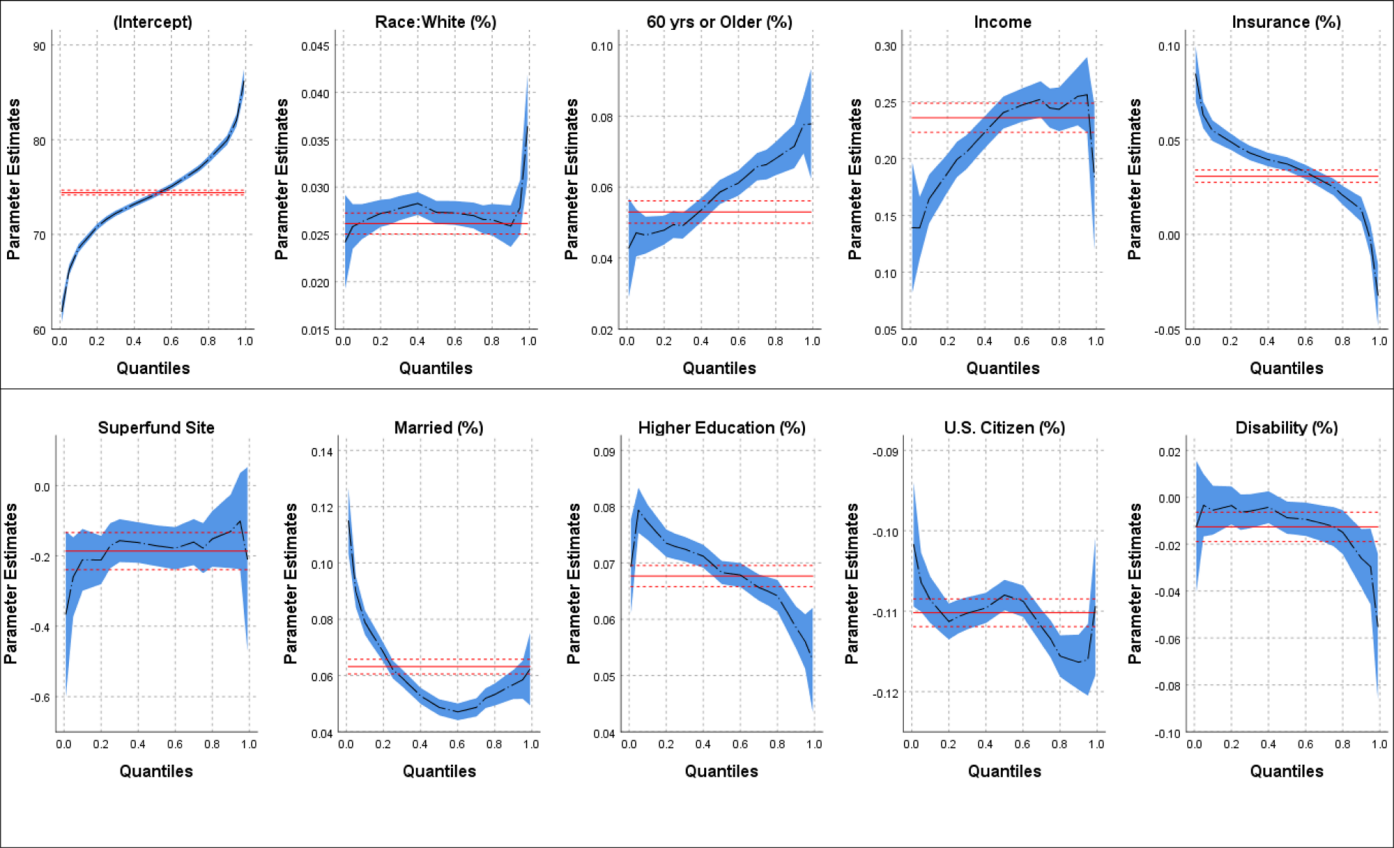
Life expectancy (LE) and sociodemographic variables. The effect of select sociodemographic variables and the presence of Superfund sites on the different percentile of LE. Only tracts with at least one Superfund site (*N* = 12,717) were chosen for the quantile regression analysis.

Figure 3 shows the results of quantile regression and the effect of sociodemographic variables and the presence of Superfund sites on different LE stratifications. The horizontal axis in Fig. 3 represents LE quantiles, while the vertical axis shows the effect of increasing one unit in the sociodemographic variables and the presence of at least one Superfund site on LE. The band around the dashed line shows the confidence intervals. The model performance, and coefficients for each of the developed model, at each quantile, is presented in Supplementary Table 5 in the SI. Supplementary Table 4 in the SI also shows that the OLS regression model has the superior performance for LE in 20% to 80% quantiles with the best performance at the median. The weakest performance was observed for the top 1% when the LE was very high, which could explain the high errors seen in Fig. 1b, c for the tracts located in the western part of the U.S. (excluding the west coast) with high LEs (Fig. 1a).

Percent white showed almost a constant effect on the different quantile of LE up to 90% percentile and a sudden jump in the higher 10% of LE. This jump could be interpreted as, among tracts with the highest LE, the impact of racial variability is more prominent compared to tracts with lower LE. However, it should be emphasized that the performance of the OLS regression model in >95% quantiles was not as good as the rest of the quantiles (Supplementary Table 4 in the SI). The percent of the population above 60 years old, and the median income in U.S. dollars showed similar patterns but over the entire LE domain; higher impacts on the upper quantiles of LE. The opposite trend was observed for education and insurance. For lower LE quantiles, the effect of these two sociodemographic factors is higher as shown in Fig. 3, and it decreases for higher LE values. This behavior means, in a tract with low LE, a 10% increase in the insurance coverage or number of people with education beyond high school diploma could lead to a rise of almost a year in LE. These effects become less influential in tracts with already high LE. The positive impact of education on LE, especially in areas with more disadvantages in sociodemographic factors, has been shown in other studies as well^44,45^. For the percent married people (married), the highest impact is in the lowest LE quantiles, while the direction of impact switched around the LE median. For the lower LE quantiles, there is a decreasing trend on the effect of married, and around the LE median, the pattern switches direction to an increasing trend. This is consistent with the effect modification findings where Superfund sites in tracts with a married ratio above the national median showed no impact, on average, on LE. For citizenship, despite multiple changes in direction, the general trend is increasing the negative effect as the LE increases. In other words, tracts with lower LEs, are less sensitive to the presence of immigrants that increase the LE. Generally, immigrants in the U.S. have longer LE compared to U.S. citizens^43^, which is compatible with the findings in both effect modification and quantile analysis. Finally, the impact of disability is minimal, similar to effect modification, among the tracts with shorter LE and becomes more influential (unfavorable) as the LE increases.

For Superfund sites, although the confidence intervals are wide, the general increasing trend could be interpreted as the lower the LE is for various reasons, the effect of Superfund sites is more severe and could magnify the negative effect of other variables. This effect becomes almost minimal in tracts with high LE as the quality of life, and the health standard is higher for people who live in these tracts^42^. In other words, among census tracts with the most disadvantage, with regards to sociodemographic variables, the negative effect of Superfund sites is more intense. As discussed earlier, for the majority of the sociodemographic variables, the effect of Superfund sites was more significant on more disadvantaged tracts, which usually have shorter LE. Importantly, in this study it was assumed that the LE data from 2010–2015 are reflective of populations residing in a given census track long enough for site exposures to have had an impact on LE. Such an assumption could mean there is likely measurement error in exposure classification. Non-differential measurement error in exposure will bias findings to the null^47^. However, it is unclear if the measurement error would be non-differential. The uncertain impacts of this residence time assumption could substantially alter findings so conducting a temporal analysis that considers the effect of Superfund site history and population mobility around these sites could potentially improve the results.

### Superfund site characteristics

The Mann–Whitney U test shows a significant difference in LE between tracts that contain Superfund site listed on NPL (*N* = 1800) and the ones not listed on the NPL (*N* = 10,917, *P*-value = 1.03E−12) with a median LE of 78.2 and 77.4 years, respectively. The OLS regression model with the NPL dummy variable showed the effect of Superfund sites could be near zero (−0.001 years) for tracts with the NPL sites as opposed to −0.217 years for tracts with sites not on NPL. Such an offsetting effect caused by being on the NPL list could be a result of the efforts conducted by EPA in redeveloping the economies of neighborhoods with a Superfund site listed on NPL, after cleanup^48^. Such activities that convert Superfund sites into a community-service area could enhance the sociodemographic conditions as well as reduce exposure. The fact that tracts with Superfund sites and with constant monitoring showed higher LE emphasizes the need for including the ~11,700 sites that are recognized as hazardous sites but are not in the current NPL in future health-related studies. In addition, the rising concerns associated with the toxicity of new emerging contaminants such as polyfluoroalkyl substances (PFAS) could add more sites to the NPL^49^.

The results of the Mann–Whitney U test showed a significant difference (*P*-value < 0.01) between tracts with active cleanup and the ones with no cleanup Superfund sites. The median LE for tracts containing active cleanup (*N* = 8619) and no cleanup (*N* = 3656) was 77.5, and 77.35 years, respectively. As noted earlier, tracts with “Unkown” status (*N* = 442) were eliminated from this analysis. The effect modification showed a minimal effect of cleanup; only 0.065 years improvement was estimated for sites with cleanup compared to the active cleanup sites. This minor change could be related to chronic and long-term exposure of people living near a Superfund site and the gap between the existence and start time of cleanup for a site. As noted before for the majority of these sites, their presence dates as far back as the 1930s and 1940 while cleanup activities did not start till the 80s^35^.

Table 3 shows the breakdown of the number of Superfund sites with different NPL and flooding statuses using the binary approach (a Superfund is considered flooded if it has an intersection with the flooding layers). The results shown in Table 3 are similar to those presented in the GAO report^30^. However, the binary definition of the flood used in GAO and other studies might be problematic. Supplementary Fig. 10 shows the number of Superfund sites at each flooding level defined as the ratio of Superfund area located in the floodplains defined by FEMA to the total area. As shown in Supplementary Fig. 10, 1043 Superfund sites (19.14% of all flooded sites and 8.69% of all studied sites, using a binary method at a radius of 322 m) showed less than 5% of their footprint are located in the floodplain. These sites could have been considered as prone to flood in the binary definition while they are excluded from the flood analysis in this study. The flooding percentage could also change by changing the radius size. A sensitivity analysis showed changing the radius from 100 m to 5000 m could change the flooding percentage by ±20%. Future work could include enhancing this analysis by using the real spatial boundaries of the Superfund sites. Using the 25% threshold, approximately 24% and 21% of Superfund sites not listed and listed on the NPL are located in a flood-prone region, respectively. A recent study showed the chance of inundation for areas situated in floodplains could range from 2 to 10 times per year across the contiguous US^50^, indicating the high probability of flooding on these sites. Superfund sites, like many chemical and industrial facilities that are vulnerable to hurricanes and flooding, could harmfully affect the life of millions of people^51^. Furthermore, greater flood-related damages have been reported in areas with property values less than $150,000^52^ (associated with lower income). Such high flood likelihood combined with the impact of being on NPL additionally supports the importance of including the ~11,700, non-NPL hazardous sites, in future studies.

**Table 3:** Number of Superfund sites based on their status with regards to the National Priorities List (NPL) and flooding.

Superfund type	Located in floodplain	Not located in floodplain	Unknown	Total
NPL^a^	800	656	224	1680
Not NPL	4650	3694	1965	10,309
Total	5450	4350	2189	11,989

^a^ Currently on the NPL or site is a part of an NPL site. The final number is different from the 1303 sites in the NPL because there are 377 locations that are part of an NPL but have a separate building/land.

For flooding classifications, the pairwise comparison showed a significant difference between sites prone to flood and the ones with minimal flood risk. The median LE for the tracts with Superfund sites prone to flood and minimal flood risk was 77.20 and 77.60 years, respectively. The OLS regression developed with the flooding dummy variable showed that tracts with a site with minimal flood risk could alter the LE by −0.034 years while the ones with flooding could amplify this effect by −0.33 years, making the final effect to −0.36 years. This difference in the effect of LE could be due to how flooding affects the fate and transport of chemicals from the contaminated sites. Flooding could introduce new exposure pathways to not only the fence-line communities but also areas located farther. Unlike fate and transport from a contaminated site under typical daily conditions, multiple routes of exposure are present during and after flooding^31,53–55^. Such a magnifying effect could be even more severe with the population growth in coastal areas combined with climate change and sea-level rise that could alter the frequency, extent, and magnitude of flooding events^33,56^. There was no significant difference in LE between tracts with sites prone to flood and minimal flood risk (*P*-value = 0.19) if a binary definition was used instead of the 25% inundation threshold.

**Fig. 4:**
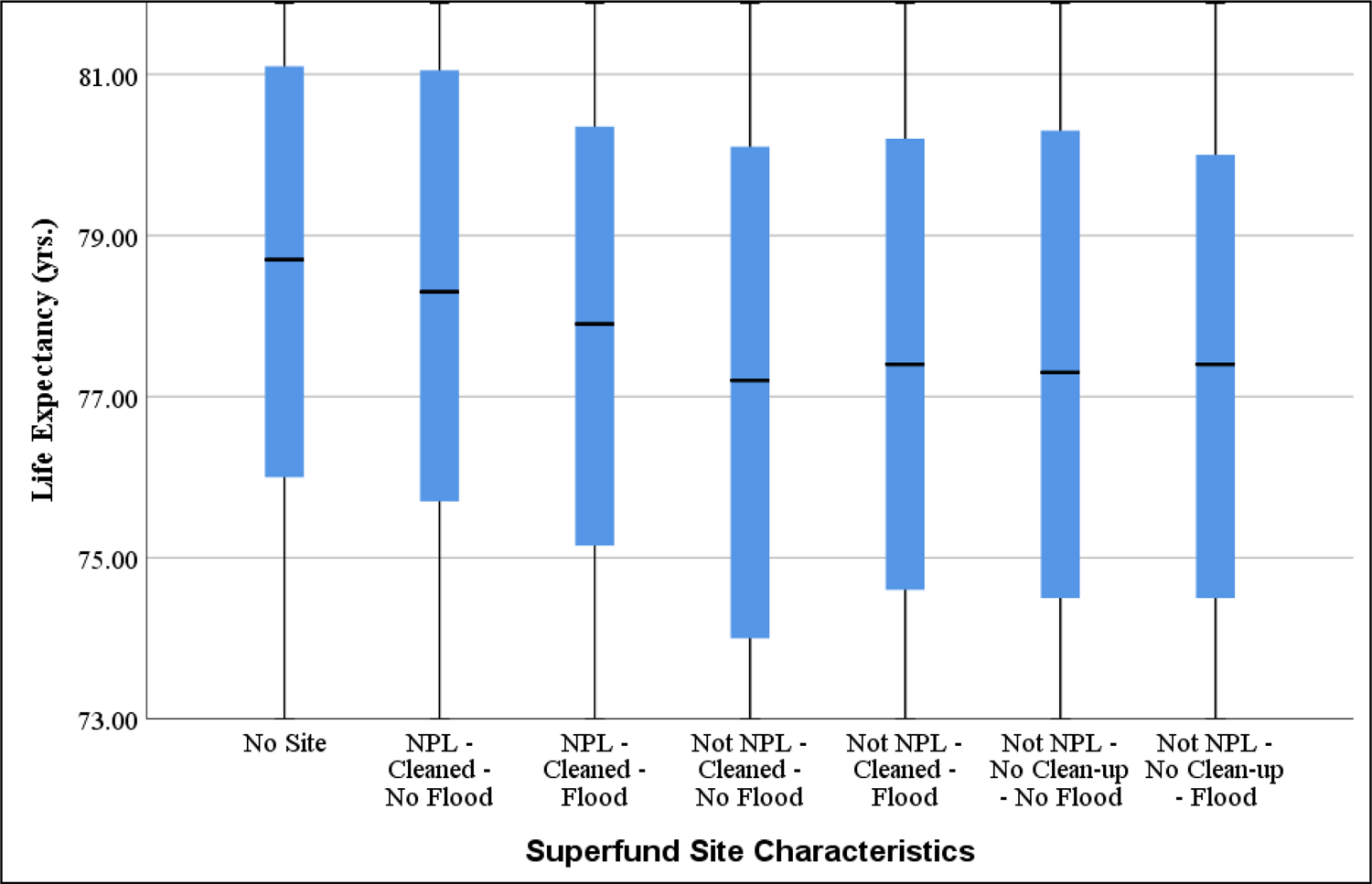
Effect of Superfund sites characteristics on life expectancy (LE). Zoomed-in box plot of LE among tracts with different Superfund site characteristics. Number of tracts were 52,509, 539, 1071, 2289, 3501, 1301, 1759 for the groups shown in the figure from left to right, respectively. Box plots indicate median (middle line), 25th, 75th percentile (box) as well as the minimum and maximum excluding outliers (whiskers). Only tracts with at least one Superfund site (*N* = 12,717) were chosen to explore the effect of Superfund characteristics.

Figure 4 shows the box plot of LE among tracts with different Superfund site characteristics. While tracts with no Superfund site showed the highest LE, the effect of being part of NPL was more critical than the other two factors (i.e., flooding and cleanup). The census tracts with sites not listed on NPL did not show that much sensitivity to flooding, unlike the ones with NPL sites. Thus, it could be concluded that the effect of being on the NPL is very influential.

In this study, statistical methods were used to provide evidence of shorter LE in association with residential proximity to a Superfund site even after accounting for measures of sociodemographic confounding. Furthermore, the research establishes that the susceptibility to Superfund site increases for census track populations that have greater sociodemographic disadvantages and that live near a Superfund site with certain characteristics (not being cleaned up, not on NPL site, and prone to flooding). Finally, using a quantile regression analysis revealed the higher effect of sociodemographic variables and Superfund site presence at census tracks with lower LE. However, the cross-sectional approach undertaken in this study lacks a temporal analysis that could potentially include tract mobility data and Superfund site history, an area for potential future research. Furthermore, in this study, a uniform exposure to Superfund sites, regardless of their contaminant of concern, physical condition, and exposure pathways, on human health was assumed. Each Superfund site requires individual investigation to more accurately estimate the harmful effect of its presence, if any, on people living in the vicinity of the site. Future studies are required to address the potential residual confounding effects not addressed in this study. Such investigation is especially relevant given the measurement error inherent in confounding measures and in the use of contextual measures.

## Methods

### Data acquisition and preparation

The National Historical Geographic Information System (NHGIS) database^57^ was used to compile 2018 census data at the census tract level. Census data on demographics, race, ethnicity, and origins, households, families, and group quarters, income, education, disability, and health insurance were downloaded for each census tract. LE data (2010-2015) were downloaded from the National Center for Health Statistics (NCHS) database^58^. For two states, Maine and Wisconsin, the LE data were not available in the database due to lack of geocoded death records; estimates were used in the current study, and the methodology applied to estimate the LE values could be found elsewhere^59^. Out of the 72,268 census tracts within the contiguous U.S., LE data was available for 65,226 tracts.

All available information on active and archived Superfund sites (as of 2019) was downloaded from the EPA Superfund Enterprise Management System database^35^. As of 2019, 1,303 sites are listed on the NPL, and 48 are proposed to be added to the NPL^35^. Supplementary Table 5 in the Supplementary Information (SI) shows the breakdown based on the site status for each state; California has the highest total number of sites with 933 sites, followed by New Jersey and New York with 754, and 646 sites, respectively. The official recognition and cleanup of Superfund sites had begun since December 11, 1980, when congress enacted the Comprehensive Environmental Response, Compensation, and Liability Act (CERCLA), commonly known as Superfund. The priority, budget, and cleanup timelines were set by the Environmental Protection Agency (EPA) via placing the most hazardous sites on the National Priorities List (NPL). According to EPA, NPL “is the list of sites of national priority among the known releases or threatened releases of hazardous substances, pollutants, or contaminants throughout the United States and its territories. The NPL is intended primarily to guide the EPA in determining which sites warrant further investigation.” While not on the NPL, for the remaining Superfund sites, EPA evaluates other cleanup options such as the Superfund Alternative Approach, State Tribal Cleanup, Other Federal Agency (FFOCA), and EPA Emergency Response and Removals.

**Fig. 5:**
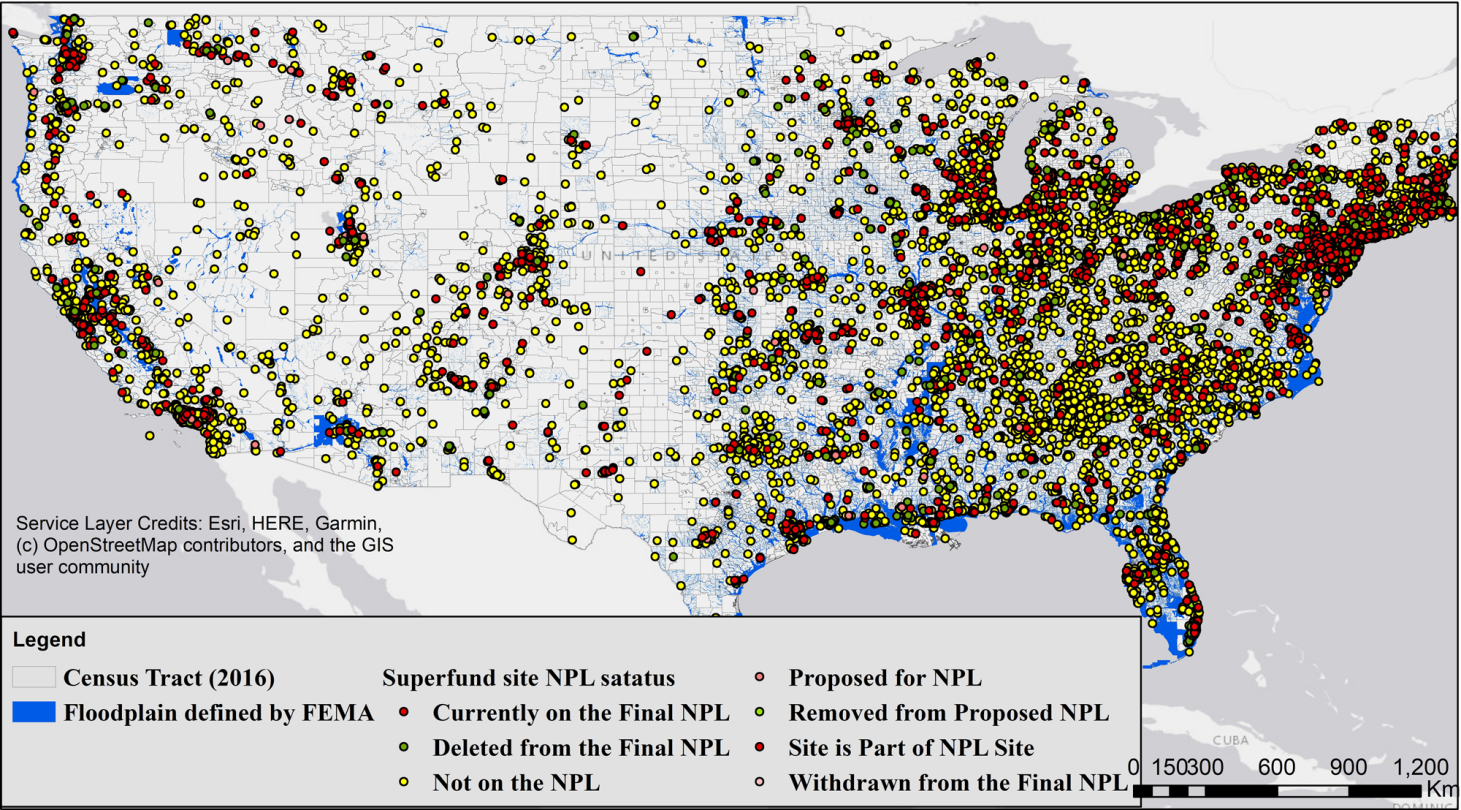
Superfund sites in the US. Location of identified Superfund sites (11,989 uniquely identified sites in the contiguous U.S.) and their status with regard to the National Priorities List (NPL). The map also shows the areas prone to flooding identified by Federal Emergency Management Agency (FEMA).

Although, as of the end of 2018, EPA has spent over 70 billion dollars of federal funding, in addition to the unknown amount of private funds^26,60^ for cleanup activities, more than 1300 sites remain on the NPL^34^ as of this writing. The main difference between an NPL site and other Superfund sites is in the amount of funding made available and the priority of cleanup activities and level of monitoring. Considering the latency between carcinogenic exposure and disease onset, the adverse effect of living near a Superfund site may only be directly observable among persons who are still living in proximity of a given site after a relatively long period of time. However, given the cross-sectional approach applied in this study, it was assumed that all people living near the sites could have been impacted since the risk of exposure in EPA’s risk assessment is based on lifetime exposure. Among the 13,093 available sites, only 1864 had latitude and longitude qualifiers. For the rest of the sites, a batch geocoding was performed based on the available addresses using the Geocodio website^61^, leading to 11,989 uniquely identified sites (points) in the contiguous U.S. as shown in Fig. 5.

Flood hazard map data were downloaded from the National Flood Hazard Layer (NFHL) database (2000–2020) maintained by the Federal Emergency Management Agency (FEMA)^62^. The flooding layers define whether an area is prone to flooding, or it has a minimal risk of flooding based on historical data and modeling efforts (predictions). The maps were individually compiled for 2485 counties and then merged in ArcMap to generate a national flood hazard map for the contiguous U.S. (see Fig. 5). FEMA flood layers, among all other data used in this study, are publicly available. To conduct the spatial analyses, a 322-m (0.2 miles) buffer was defined around each of the sites; this value representing a risk-analysis distance measure used in prior studies^30^. A sensitivity analysis was also conducted using smaller and larger radii. Using the floodplain areas provided by FEMA, the Superfund sites were categorized into prone to flood (fully or partially located within floodways, 100, or 500-year floodplains), minimal flood risk (area of minimal flood hazard, or area with reduced flood risk due to levee), or unknown (area not included, or no available by FEMA). The percent of each site located in floodplains, defined by using the “intersect” tool in ArcMap, was used as a criterion for the determination; a site with 25% or more of its area located in floodplains was categorized as a site that is prone to flood.

Another classification was considered based on the presence of Superfund sites on the NPL^35^. A site that is currently on the final NPL, or is part of an NPL site was considered “NPL” while the rest were classified as “Not NPL.” In other words, in this study, all sites that are currently on the NPL were considered as one class and the rest, including proposed or waiting to be on the list, removed from the list, had other EPA cleanup options, and sites that are not overseen by EPA, were placed in another class (“Not NPL”). The idea behind this classification was to investigate if there is any significant difference in the effect of sites belonging to the two categories on the LE. Further analysis beyond NPL-not NPL classification is beyond the scope of this study. Finally, the cleanup status was considered as “active cleanup” for NPL sites and any non-NPL sites with a cleanup status indicating cleanup activity; “no cleanup” for non-NPL sites under assessment or review; and “unknown” for sites with no specified status. The Spatial Join tool in ArcMap was applied to layer the Superfund site information into the Census tracts. In cases where two or more Superfund sites were found within a tract, the priority classification was given to “prone to flood,” “NPL,” and “no cleanup” for flooding, site type, and cleanup status, respectively. In all statistical analyses, the “unknown” groups were eliminated.

### Statistical modeling of the Superfund and LE relationship

A list of the sociodemographic variables used in the current study (population above 60 years old, median income and income per capita in U.S. dollars, population below the poverty line, race, population with at least one disability, marriage status, population with at least one health insurance plan, population with education beyond high school diploma, U.S. citizenship) is provided in Supplementary Table 1 in the SI. A bivariate Spearman’s rank correlation analysis was conducted to investigate the significance of the correlation among sociodemographic variables, the presence of Superfund sites, and LE. The results of the correlations were used to choose the input variables for use in the developed statistical models. Kolmogorov–Smirnov normality test showed that none of the census datasets are normal. However, the LE distribution was very close to the bell-shaped normal distribution. Thus, non-parametric (NP) statistical tests were applied for all variables in the study except the LE for which both parametric and NP tests were used.

In addition to the conventional statistical tests for comparison, two different approaches were developed to answer the first two research questions raised in the Introduction. The first approach intended to investigate the possibility; a matching technique was used to demonstrate that the presence of a Superfund site (NPL or non-NPL) in a census tract could actually make a difference in terms of LE independently. The second approach intended to determine the magnitude of the effect by developing regression models that were used to quantify the potential effect of the presence of a Superfund site (NPL or non-NPL) on the LE in the context of other sociodemographic determinants. The performance of the developed regression model in this study was tested against a non-linear machine-learning (ML) algorithm (Random Forests) to examine the validity of the regression model. The following sections describe the two approaches in more detail.

### Matching technique

Matching methods have been used in statistical modeling to estimate causal effects using observational data in the context of “treated” and “untreated” populations^63^. For the purposes of the current study, the matching technique was implemented by considering tracts with Superfund sites as the “treated group”, and comparing the LE in treated and untreated groups (with no Superfund site). To clarify the analogy, these two groups will be called “exposed” and “unexposed” for the rest of the paper. Due to the continuous nature and high geospatial variability of the sociodemographic matching variables (income, race, and so on as listed above), a non-exact matching was performed based on spatial proximity. In this approach, the exposed observation (a census tract with a Superfund site) was compared with its nearest neighbor. Thus, census tracts that contain at least one Superfund site within their boundaries were marked as exposed using the spatial join tool in ArcMap. The LE in an exposed tract was compared to the median of LEs in vicinity tracts without any Superfund sites (unexposed). For this purpose, tracts with a common border with the exposed tracts were extracted using ArcMap and exported to Microsoft Excel. A pivot table with customized formulation was then used to calculate the median LE and other census variables for the neighboring tracts. In other words, for each exposed tract, the LE and other sociodemographic variables were compared to the corresponding values of estimated conditions of its immediate neighbors.

Independent-Samples Mann–Whitney U test was performed to find differences, if any, among exposed and median of unexposed tracts for LE and other sociodemographic variables. For LE, an independent two-tailed t-test was also performed. In order to ensure that the potential observed difference in LE is not due to the innate differences in LE among neighboring census tracts, the matching analyses were repeated for all tracts without consideration of the presence or absence of Superfund sites within them.

### Modeling of the magnitude of impact of Superfund sites on LE

A linear regression model using OLS was developed to quantify the potential impact of the presence of a Superfund site in a census tract on the LE in the tract. The impact of Superfund sites was introduced to the model via a binary variable with 0 and 1 representing the absence and presence of at least one Superfund site, respectively. An initial OLS regression model was developed with just the Superfund site as the independent variable and LE as the dependent variable. Populations living near hazardous waste sites generally have a greater sociodemographic disadvantage^22^ (confirmed by a Mann–Whitney U test) and, as a result, have poorer health. Thus, other sociodemographic variables (selected based on the pairwise correlations) were then added in a stepwise manner in the order of their correlation magnitude with the LE (a total of nine OLS regression models). Including all of the sociodemographic variables, especially income, in the final OLS regression model was undertaken to avoid their confounding effect. Furthermore, using such an approach makes it possible to quantify the impact of the presence of Superfund sites on LE while considering other influential sociodemographic variables. All sociodemographic variables except income were input to the model as a percent (0–100%), and income was entered into the model as a multiple of $10,000 (i.e., 3.7 instead of $37,000).

### Random Forests (RF) machine learning algorithm

To confirm the validity of an OLS regression algorithm in the study, a more sophisticated model was developed to compare its general performance in predicting LE with the OLS regression model. Given that the relationship between LE and the selected sociodemographic variables is most likely non-linear, it is important to use a non-linear model to explore the extent of variability in the LE that can be explained by the variables. The difference in the residual standard error between the linear and non-linear model could reveal how much improvement in LE prediction could be achieved by using the non-linear model. A predictive Random Forests (RF) algorithm, ‘treebagger”, a built-in MATLAB function, was used with the number of trees = 100 and the minimum number of observations per tree leaf= 20 to predict LE. The RF algorithm was chosen from many other existing algorithms because of its extensive capability in dealing with big data and a large number of variables, simplicity, high accuracy, and relatively short computation time^64,65^. Out of the 65,226-census tracts with data, 50,000 were chosen randomly for the purpose of training, and the rest were used for validation.

It is noted that the outcome of the non-linear model cannot be easily interpreted with regards to the effect of each input variable, thus, if the performance of RF in predicting the LE is similar to the one from the OLS regression, it would justify the use of the OLS model. It is preferable to use the linear regression in analyzing data due to more interpretability of the results.

### Identifying the most vulnerable populations

In this section, an effect modification approach was developed to investigate the relationship between the presence of a Superfund site and the sociodemographic variables and how this relationship could affect the LE (research question 3). Furthermore, a quantile analysis was undertaken to breakdown the effect of sociodemographic variables and Superfund presence among different LE stratifications. Similarly, and for research question 4, effect modification was performed to assess how Superfund sites with different properties, flooding, NPL, and cleanup activities, may affect the LE differently.

### Effect modification and quantile analysis

Effect modification analyses were undertaken to investigate whether the presence of Superfund sites has a different effect on LE among different subgroups (stratifications) of sociodemographic variables. For each variable, a binary dummy variable was defined as the product of Superfund presence (0 or 1) and being above (1) or below (0) the median value of the corresponding sociodemographic variable; a separate OLS regression was performed for each variable:

2 $${\mathrm{LE}} = \beta _0 + \mathop {\sum }\limits_{{{i}} = 1}^{{n}} \beta _iX_i + \beta _jD_j$$

where *β*_0_ is the OLS regression constant, *β*_*i*_ is the regression coefficient for the Superfund sites and sociodemographic variables, n is the total number of variables in the regression, *X*_*i*_ is the sociodemographic variable, *D*_*j*_ is the binary dummy variable explained earlier (with a value of 0 or 1), which is the product of Superfund presence and being above or below the median, and *β*_*j*_ is the regression coefficient for the binary dummy variable. A total of eight OLS regression models (*j* = 1–8) were built for effect modification. The effect modification analysis was conducted by comparing *β*_*i*_ and *β*_*i*_ + *β*_*j*_ for each of the eight developed OLS regression models.

While effect modification investigates the varying effects of Superfund site presence among different subgroups (based on sociodemographic variables), assessing the impact of Superfund site on different stratifications of the dependent variable (i.e., LE) could also be informative. Furthermore, the quantile regression analyses were performed to investigate additional aspects of the relationship between LE and the selected independent sociodemographic variables. Quantile regression addresses the non-linear nature of the problem as well as the skewness inherent in some of the sociodemographic variables that cannot be addressed using the OLS regression^66^.

### The effect of Superfund site characteristics on LE

Only tracts with at least one Superfund site (*N* = 12,717) were chosen to explore the effect of Superfund characteristics. Independent-Samples Kruskal–Wallis Test and Mann–Whitney U test were applied to find a significant difference in LE, if any, among tracts with Superfund sites with different properties in flooding, NPL status, and cleanup conditions. A similar approach to the effect modification was undertaken (using all tracts with available data) to assess the effect of Superfund characteristics on LE. For each characteristic, a dummy variable was defined as the product of Superfund presence and the corresponding binary membership defined based on the site characteristics (e.g., being in a flood-prone area, remediated, or part of NPL were presented as 1 in the multiplication). A total of three separate OLS regressions were developed to capture the aforementioned effects.

### Reporting summary

Further information on research design is available in the Nature Research Reporting Summary linked to this article.

## Supplementary information

Supplementary Information

Reporting Summary

## Data Availability

All data used in this study are publicly available through the provided citations in the text. The following sources of data were used in this work: 1. Sociodemographic data was downloaded from the National Historical Geographic Information System (NHGI) database (10.18128/D050.V14.0) 2. Life expectancy data were downloaded from the National Center for Health Statistics (NCHS) database (https://www.cdc.gov/nchs/nvss/usaleep/usaleep.html). For two states, Maine and Wisconsin, the LE data were not available in the database due to lack of geocoded death records 3. All available information on active and archived Superfund sites was downloaded from the EPA’s Superfund Enterprise Management System database (https://cumulis.epa.gov/supercpad/cursites/srchsites.cfm) 4. Flood hazard map data were downloaded from the National Flood Hazard Layer (NFHL) database maintained by the Federal Emergency Management Agency (FEMA) (https://www.floodmaps.fema.gov/NFHL/status.shtml.) Figure 1 has raw data from sources 1 and 3 (census tract shapefile, and Superfund NPL status), Fig. 2a has raw data from source 2, and Supplementary Figs. 1–8 in the Supplementary Information (SI) has raw data from source 1. All generated results (including the raw data) in both tabulated and shapefile formats have been deposited in the Open Science Framework (OSF) under the name “KiaghadiEtAl_Nature_Communications_DATA” and are accessible through^67^.

## Code availability

The codes developed to perform the Random Forest algorithm could be found in ref. ^68^.
