# Supplementary material for: The presence of Superfund sites as a determinant of life expectancy in the United States
Source: Nat Commun. 2021 Apr 13;12:1947. doi: 10.1038/s41467-021-22249-2 (PMC8044172; doi:10.1038/s41467-021-22249-2)
Supplement: Supplementary file 1 — Supplementary Information [file 41467_2021_22249_MOESM1_ESM.pdf]

The Presence of Superfund Sites as a Determinant of Life Expectancy in the United States

Amin Kiaghadi [1,2], Hanadi S. Rifai[1*], Clint N. Dawson[2]

[1] Civil and Environmental Engineering, University of Houston, Houston, TX 77204-4003, USA

[2] Oden Institute for Computational Engineering and Sciences, University of Texas at Austin, Austin, TX 78712, USA

[*] Corresponding author: rifai@uh.edu

Table 1. A summary of statistics for LE and various sociodemographic variables in all census tracts, tracts with at least one Superfund site, and tracts with no site. For all variables except life expectancy (year), income per capita, and median income (multiple of $10,000) the unit is percent.

Statistics	Life expectancy	Above60[1]	White	African-American	Hispanic	Below poverty line	Income per capita	Median income	Health insurance[2]	Above65 with insurance	Married	Post high school degree[4]	U.S. Citizenship	Disability[4]
All data														
N	65,226													
Minimum	56.30	1.00	0.00	0.00	0.00	0.00	0.29	0.68	3.00	0.00	2.89	4.48	13.22	0.00
Maximum	97.50	92.00	100.00	100.00	100.00	88.00	22.71	25.00	95.00	91.00	85.93	99.49	100.00	42.90
Median	78.50	21.00	82.00	4.00	7.00	13.00	2.62	5.26	71.00	29.00	51.06	56.77	92.76	7.52
Mean	78.28	21.46	73.06	13.86	16.37	15.66	2.95	5.92	70.80	29.64	49.67	57.56	87.47	8.02
Std. Error of Mean	0.02	0.03	0.10	0.09	0.08	0.04	0.01	0.01	0.03	0.04	0.05	0.07	0.05	0.01
Std. Deviation	4.00	8.33	25.13	22.08	21.59	11.49	1.51	2.92	8.72	11.05	12.19	17.45	13.66	3.36
No Superfund site														
N	52,509													
Minimum	56.30	2.00	0.00	0.00	0.00	0.00	0.34	0.68	3.00	0.00	2.89	4.48	13.22	0.00
Maximum	97.50	92.00	100.00	100.00	100.00	88.00	22.71	25.00	95.00	91.00	85.93	99.49	100.00	37.67
Median	78.70	21.00	82.00	4.00	7.00	12.00	2.67	5.38	72.00	29.00	51.63	57.89	92.44	7.53
Mean	78.50	21.57	73.55	13.15	16.63	15.07	3.01	6.05	70.98	29.66	50.28	58.42	87.12	8.04
Std. Error of Mean	0.02	0.04	0.11	0.09	0.09	0.05	0.01	0.01	0.04	0.05	0.05	0.08	0.06	0.01
Std. Deviation	3.91	8.50	24.72	21.30	21.68	11.09	1.52	2.94	8.72	11.05	11.99	17.51	13.88	3.36
At least one Superfund site														
N	12,717													
Minimum	58.10	1.00	0.00	0.00	0.00	0.00	0.29	0.89	7.00	0.00	3.26	4.51	17.07	0.00
Maximum	93.60	85.00	100.00	100.00	100.00	84.00	19.49	25.00	94.00	83.00	79.93	99.43	100.00	42.90
Median	77.50	21.00	81.00	5.00	6.00	15.00	2.42	4.80	71.00	29.00	48.54	52.53	94.03	7.47
Mean	77.36	21.02	71.06	16.80	15.33	18.11	2.74	5.41	70.06	29.55	47.17	54.04	88.91	7.96
Std. Error of Mean	0.04	0.07	0.24	0.22	0.19	0.11	0.01	0.02	0.08	0.10	0.11	0.15	0.11	0.03
Std. Deviation	4.23	7.55	26.64	24.83	21.20	12.71	1.44	2.79	8.65	11.05	12.68	16.76	12.59	3.33

[1] Percent of the population above 60 years old, [2] Percent of the population with at least one health insurance plan, [3] Percent of the population with education beyond a high school diploma, [4] Percent of the population with at least one disability

Table 2. Spearman's rank correlation analysis

Variable	Life expectancy	Above60	White	African-American	Hispanic	Below poverty line	Income per capita	Median income	Health insurance	Above65 with insurance	Superfund site	Married	Post high school degree	U.S. Citizenship	Disability
Life expectancy	1.000	0.153	0.218	-0.360	0.060	-0.590	0.635	0.675	0.404	-0.020	-0.100	0.466	0.573	-0.280	0.022
Above60[1]	0.153	1.000	0.450	-0.310	-0.420	-0.260	0.309	0.104	-0.220	-0.150	-0.010	0.347	0.127	0.371	0.057
White	0.218	0.450	1.000	-0.760	-0.400	-0.390	0.327	0.262	0.080	-0.190	-0.020	0.560	0.155	0.479	-0.070
African-American	-0.360	-0.310	-0.760	1.000	0.122	0.364	-0.310	-0.320	-0.130	0.086	0.051	-0.540	-0.160	-0.150	0.064
Hispanic	0.060	-0.420	-0.400	0.122	1.000	0.210	-0.200	-0.060	-0.090	0.253	-0.030	-0.260	-0.130	-0.740	0.012
Below Poverty line	-0.590	-0.260	-0.390	0.364	0.210	1.000	-0.800	-0.850	-0.490	0.112	0.096	-0.640	-0.650	-0.010	-0.040
Income per capita	0.635	0.309	0.327	-0.310	-0.200	-0.800	1.000	0.866	0.533	-0.110	-0.090	0.526	0.837	-0.060	0.052
Medina income	0.675	0.104	0.262	-0.320	-0.060	-0.850	0.866	1.000	0.612	-0.060	-0.100	0.610	0.738	-0.160	0.071
Health insurance[2]	0.404	-0.220	0.080	-0.130	-0.090	-0.490	0.533	0.612	1.000	0.094	-0.040	0.279	0.540	-0.100	0.024
Above65 with insurance[3]	-0.020	-0.150	-0.190	0.086	0.253	0.112	-0.110	-0.060	0.094	1.000	**0.005**	-0.110	-0.090	-0.230	0.018
Superfund site[4]	-0.100	-0.010	-0.020	0.051	-0.030	0.096	-0.090	-0.100	-0.040	**0.005**	1.000	-0.090	-0.100	0.056	0.008
Married	0.466	0.347	0.560	-0.540	-0.260	-0.640	0.526	0.610	0.279	-0.110	-0.090	1.000	0.352	0.180	-0.017
Post high school degree	0.573	0.127	0.155	-0.160	-0.130	-0.650	0.837	0.738	0.540	-0.090	-0.100	0.352	1.000	-0.110	0.029
U.S. Citizenship	-0.280	0.371	0.479	-0.150	-0.740	-0.010	-0.060	-0.160	-0.100	-0.230	0.056	0.180	-0.110	1.000	-0.045
Disability[5]	0.022	0.057	-0.070	0.064	0.012	-0.040	0.052	0.071	0.024	0.018	0.008	-0.010	0.029	-0.040	1.000

[1] Percent of the population above 60 years old, [2] Percent of the population with at least one health insurance plan, [3] Percent of the population with education beyond a high school diploma, [4] Percent of the population with at least one disability , Bold = not significant. Grey shaded cell = significant with alpha=0.05, the rest are significant with alpha=0.01

Table 3. Model performance and obtained coefficients for the 8 manual stepwise OLS regression models

1					
R	R^2	Adjusted R^2	RMSE	df	F
0.11	0.01290	0.01289	3.97	1	852.43
Variable	Unstandardized Coefficients	Std. Error	Standardized Coefficients	t	Sig.
(Constant)	78.505	0.017		4529.513	0.000
Superfund site	-1.146	0.039	-0.114	-29.196	0.000

2					
R	R^2	Adjusted R^2	RMSE	df	F
0.61	0.37259	0.37257	3.17	2	19366.82
Variable	Unstandardized Coefficients	Std. Error	Standardized Coefficients	t	Sig.
(Constant)	73.526	0.029		2516.112	0.000
Superfund site	-0.626	0.031	-0.062	-19.932	0.000
Income	0.824	0.004	0.602	193.371	0.000

3					
R	R^2	Adjusted R^2	RMSE	df	F
0.63	0.39810	0.39807	3.10	3	14379.12
Variable	Unstandardized Coefficients	Std. Error	Standardized Coefficients	t	Sig.
(Constant)	71.808	0.043		1653.195	0.000

7					
R	R^2	Adjusted R^2	RMSE	df	F
0.73	0.53800	0.53800	2.72	7	10853.97
Variable	Unstandardized Coefficients	Std. Error	Standardized Coefficients	t	Sig.
(Constant)	75.584	0.120		631.148	0.000
Superfund site	-0.182	0.027	-0.018	-6.700	0.000
Income	0.222	0.007	0.162	33.742	0.000
Education	0.076	0.001	0.331	81.446	0.000
Married	0.074	0.001	0.227	57.324	0.000
Health insurance	0.006	0.002	0.014	4.101	0.000
Citizenship	-0.104	0.001	-0.356	-119.983	0.000
White	0.028	0.001	0.176	49.486	0.000

8					
R	R^2	Adjusted R^2	RMSE	df	F
0.74	0.54600	0.54600	2.69	8	9793.48
Variable	Unstandardized Coefficients	Std. Error	Standardized Coefficients	t	Sig.
(Constant)	74.305	0.125		594.938	0.000
Superfund site	-0.185	0.027	-0.018	-6.875	0.000

Superfund site	-0.538	0.031	-0.053	-17.455	0.000
Income	0.598	0.006	0.437	99.795	0.000
Education	0.053	0.001	0.230	52.567	0.000
**4**					
R	R^2	Adjusted R^2	RMSE	df	F
0.66	0.43556	0.43553	3.00	4	10065.69
Variable	Unstandardized Coefficients	Std. Error	Standardized Coefficients	t	Sig.
(Constant)	68.734	0.063		1093.387	0.000
Superfund site	-0.398	0.030	-0.039	-13.319	0.000
Income	0.378	0.007	0.276	56.401	0.000
Education	0.061	0.001	0.266	62.127	0.000
Married	0.078	0.001	0.238	65.797	0.000
**5**					
R	R^2	Adjusted R^2	RMSE	df	F
0.72	0.52074	0.52071	2.77	5	14173.17
Variable	Unstandardized Coefficients	Std. Error	Standardized Coefficients	t	Sig.
(Constant)	68.737	0.116		592.398	0.000
Superfund site	-0.398	0.030	-0.039	-13.317	0.000
Income	0.378	0.007	0.276	53.937	0.000
Education	0.061	0.001	0.266	60.707	0.000

Income	0.234	0.007	0.171	35.833	0.000
Education	0.068	0.001	0.296	71.079	0.000
Married	0.063	0.001	0.194	47.745	0.000
Health insurance	0.030	0.002	0.066	18.239	0.000
Citizenship	-0.110	0.001	-0.376	-125.166	0.000
White	0.026	0.001	0.166	46.821	0.000
Above60	0.052	0.002	0.109	33.095	0.000
**9**					
R	R^2	Adjusted R^2	RMSE	df	F
0.74	0.54350	0.54344	2.70	8	9705.65
Variable	Unstandardized Coefficients	Std. Error	Standardized Coefficients	t	Sig.
(Constant)	74.409	0.128		583.145	0.000
Superfund site	-0.186	0.027	-0.018	-6.926	0.000
Income	0.236	0.007	0.172	36.034	0.000
Education	0.068	0.001	0.295	70.813	0.000
Married	0.063	0.001	0.193	47.470	0.000
Health insurance	0.031	0.002	0.067	18.346	0.000
Citizenship	-0.110	0.001	-0.376	-125.207	0.000
White	0.026	0.001	0.164	46.313	0.000
Above60	0.053	0.002	0.110	33.330	0.000

Married	0.078	0.001	0.238	65.665	0.000
Health insurance	0.000	0.002	0.000	-0.027	0.978
**6**					
R	R^2	Adjusted R^2	RMSE	df	F
0.72	0.52100	0.52100	2.77	6	11811.51
Variable	Unstandardized Coefficients	Std. Error	Standardized Coefficients	t	Sig.
(Constant)	74.844	0.121		618.405	0.000
Superfund site	-0.173	0.028	-0.017	-6.265	0.000
Income	0.194	0.007	0.142	29.138	0.000
Education	0.081	0.001	0.353	85.787	0.000
Married	0.108	0.001	0.328	95.226	0.000
Health insurance	0.002	0.002	0.005	1.438	0.150
Citizenship	-0.090	0.001	-0.306	-107.677	0.000

Disability	-0.013	0.003	-0.011	-3.968	0.000

Table 4. Model performance, and coefficients for each of the developed models at each quantile

Model Statistics																					
Statistics	Quantile																				
	1%	2%	3%	4%	5%	10%	20%	25%	30%	40%	50%	60%	70%	75%	80%	90%	95%	96%	97%	98%	99%
R^2	0.365	0.375	0.378	0.379	0.380	0.383	0.379	0.375	0.371	0.362	0.350	0.334	0.312	0.299	0.286	0.254	0.226	0.217	0.206	0.191	0.171
MAE	6.32	5.48	4.96	4.59	4.31	3.48	2.69	2.47	2.30	2.11	2.05	2.11	2.31	2.49	2.74	3.68	4.69	5.01	5.47	6.10	7.14
Parameter	Coefficients																				
Intercept	61.85	63.05	64.20	65.27	66.27	68.54	70.80	71.60	72.22	73.20	74.08	75.07	76.35	77.00	77.92	79.97	82.09	82.37	83.20	84.43	86.18
White	0.024	0.026	0.025	0.026	0.026	0.026	0.027	0.027	0.028	0.028	0.027	0.027	0.027	0.027	0.027	0.026	0.028	0.029	0.030	0.032	0.036
Above60	0.043	0.047	0.051	0.048	0.047	0.046	0.048	0.049	0.049	0.054	0.059	0.061	0.066	0.066	0.068	0.072	0.078	0.082	0.088	0.087	0.078
Income	0.139	0.148	0.142	0.139	0.139	0.164	0.187	0.199	0.205	0.223	0.241	0.247	0.252	0.244	0.243	0.255	0.256	0.261	0.259	0.242	0.183
Insurance	0.085	0.081	0.077	0.069	0.063	0.055	0.049	0.046	0.043	0.039	0.037	0.033	0.028	0.025	0.021	0.013	-0.003	-0.003	-0.007	-0.017	-0.032
Superfund	-0.365	-0.362	-0.270	-0.277	-0.260	-0.211	-0.212	-0.171	-0.157	-0.162	-0.171	-0.178	-0.161	-0.178	-0.152	-0.130	-0.101	-0.125	-0.093	-0.156	-0.209
Married	0.115	0.100	0.094	0.092	0.090	0.079	0.068	0.062	0.059	0.053	0.049	0.047	0.049	0.052	0.053	0.057	0.058	0.058	0.055	0.058	0.062
Education	0.069	0.072	0.077	0.078	0.079	0.077	0.074	0.073	0.072	0.071	0.068	0.068	0.066	0.065	0.064	0.059	0.056	0.054	0.052	0.051	0.053
Citizen	-0.102	-0.101	-0.104	-0.104	-0.106	-0.109	-0.111	-0.111	-0.110	-0.110	-0.108	-0.109	-0.112	-0.113	-0.116	-0.116	-0.116	-0.115	-0.115	-0.113	-0.109
Disability	-0.012	0.006	0.000	0.002	-0.003	-0.006	-0.004	-0.006	-0.006	-0.004	-0.009	-0.009	-0.011	-0.013	-0.015	-0.026	-0.030	-0.034	-0.041	-0.054	-0.055

R^2= Coefficient of determination, MAE= Mean absolute error

Table 5. Number of Superfund sites based on their status relative to the National Priorities List (NPL) for different states in the United States sorted by highest total number of sites

State	Currently on the Final NPL	Deleted from the Final NPL	Not on the NPL	Proposed to NPL	Removed from the Proposed NPL	Site is Part of an NPL Site	Withdrawn from the Final NPL	Total
California	98	14	793	2	6	20		933
New Jersey	114	35	592	1	1	11		754
New York	84	33	522	2	1	4		646
Michigan	67	19	523	2	6	3		620
Ohio	39	7	542	5	2	1		596
Illinois	45	6	505	5	4	14		579
Florida	53	27	471	1	3	4		559
Georgia	16	5	445	1	1	1		469
Texas	54	13	331	1		6		405
Indiana	41	10	326	1	3	9	1	391
North Carolina	38	4	342			4	2	390
Massachusetts	31	8	318	1		19		377
Pennsylvania	91	33	227	2		3		356
Tennessee	19	5	284		1		1	310
Alabama	12	3	284	2		1		302
Missouri	34	5	205		2	41		287
Connecticut	13	4	239	1		24		281
South Carolina	26	6	223			2		257
Wisconsin	36	8	208	1	1			254
Washington	46	18	104			59		227
Kentucky	13	7	180					200
Colorado	20	3	137	1	1	14		176
Virginia	30	3	138			1		172
Arizona	9	3	149			6		167
New Mexico	16	4	134			1		155
Utah	13	8	116	3	1	13		154
Louisiana	13	12	120	3		4		152
Minnesota	24	22	100					146

State	Currently on the Final NPL	Deleted from the Final NPL	Not on the NPL	Proposed to NPL	Removed from the Proposed NPL	Site is Part of an NPL Site	Withdrawn from the Final NPL	Total
Oregon	13	5	111	1	1	14		145
Mississippi	9	4	109	1	1			124
Rhode Island	12	1	90			20		123
Oklahoma	8	7	93	1		4		113
Maryland	19	4	86	1		1	1	112
Nebraska	17	1	59			31		108
West Virginia	10	3	91			2		106
Kansas	12	5	81	1	1	5		105
Iowa	11	11	60	1	2	9	1	95
Idaho	6	3	61	3		14		87
Montana	17		62	1	2	1		83
New Hampshire	20	1	49	1		6		77
Arkansas	9	8	52		1	2		72
Nevada	1		60	1				62
Delaware	15	7	34	1			1	58
Maine	12	4	39			2		57
Vermont	12	2	32			1		47
South Dakota	2	2	29					33
Wyoming	2	1	30					33
District of Columbia	1		25					26
North Dakota		2	6					8

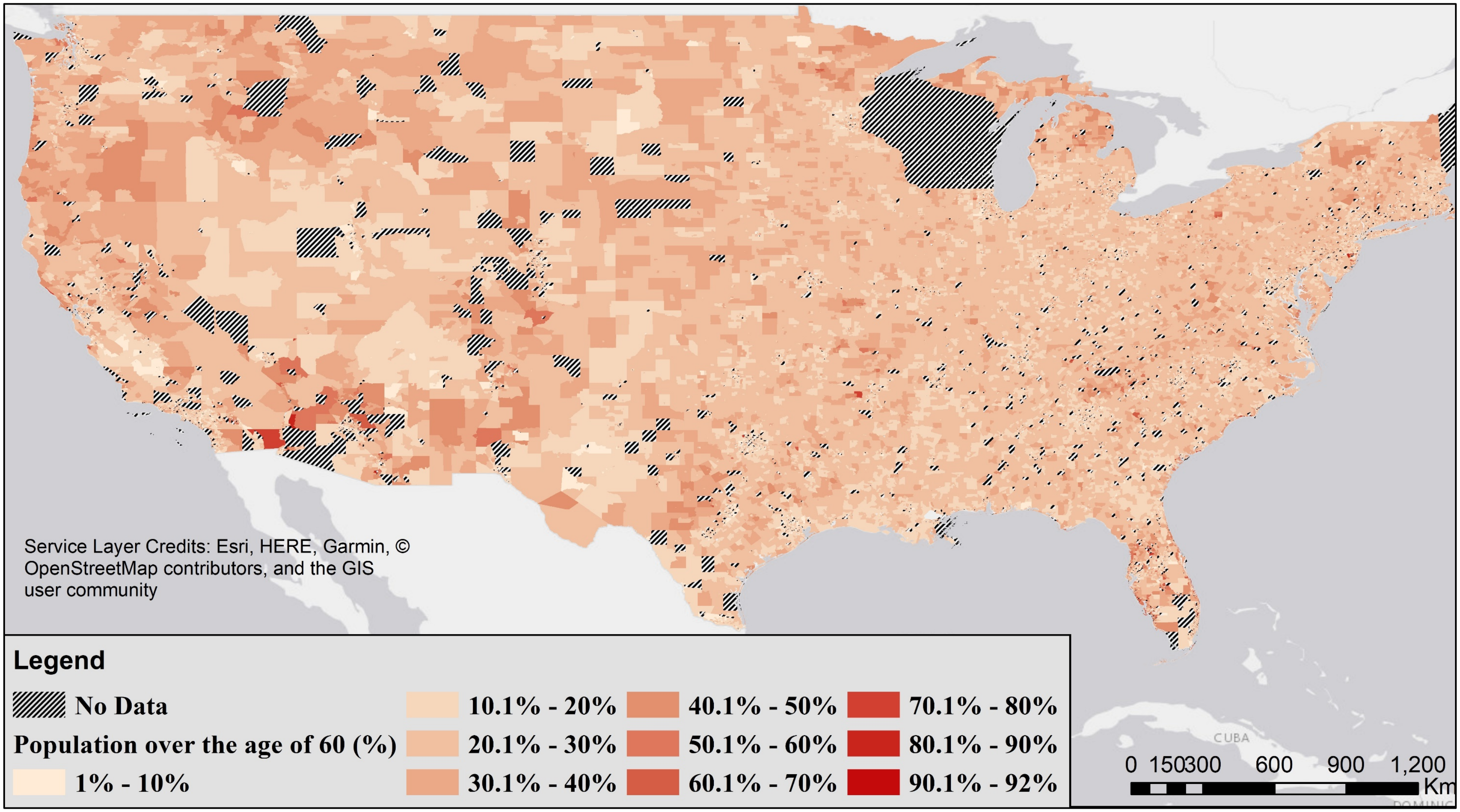

Figure 1. Percent of the population above 60 years old (variable name: above 60) in all census tracts with available data

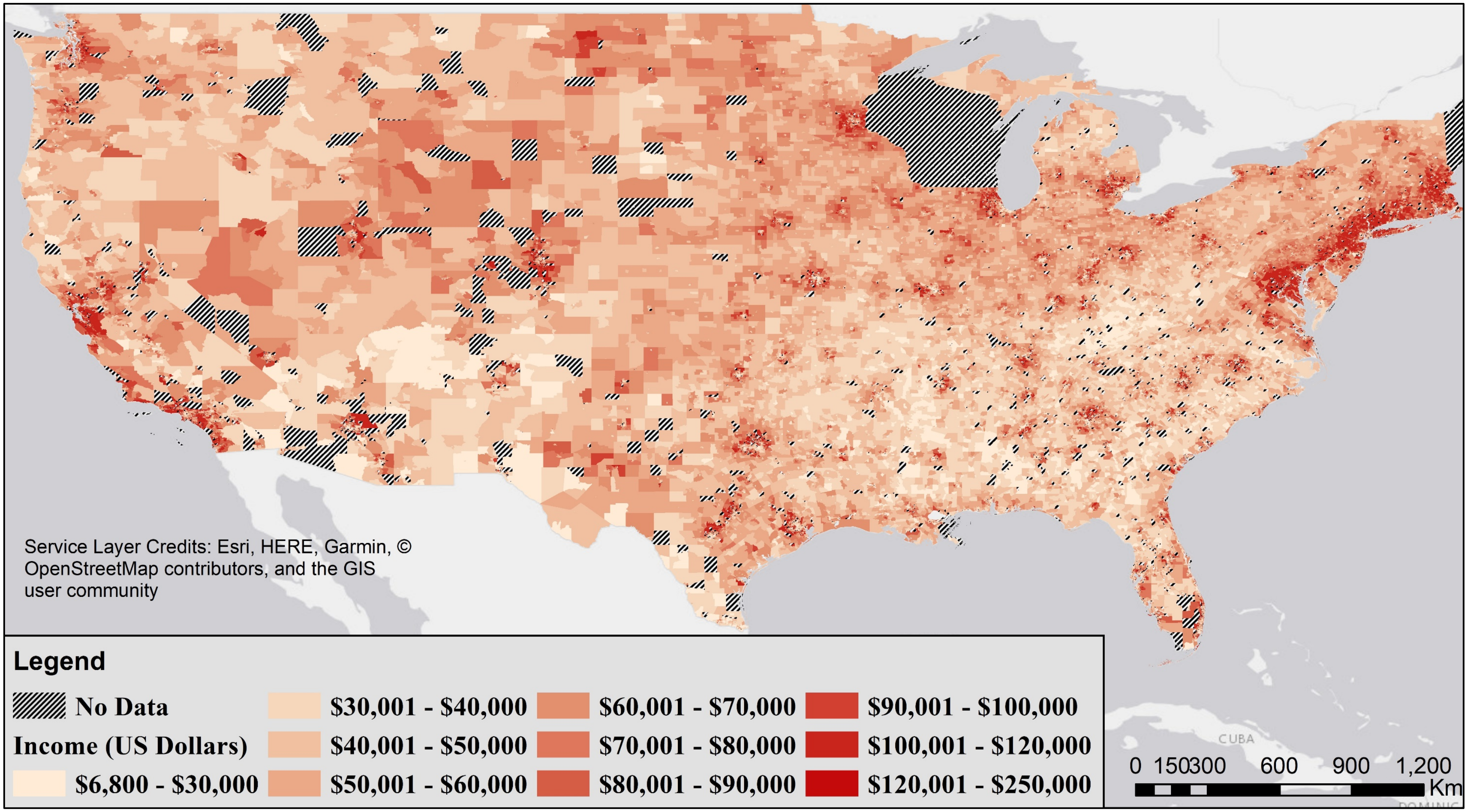

Figure 2. The median income in U.S. dollars (variable name: income) in all census tracts with available data

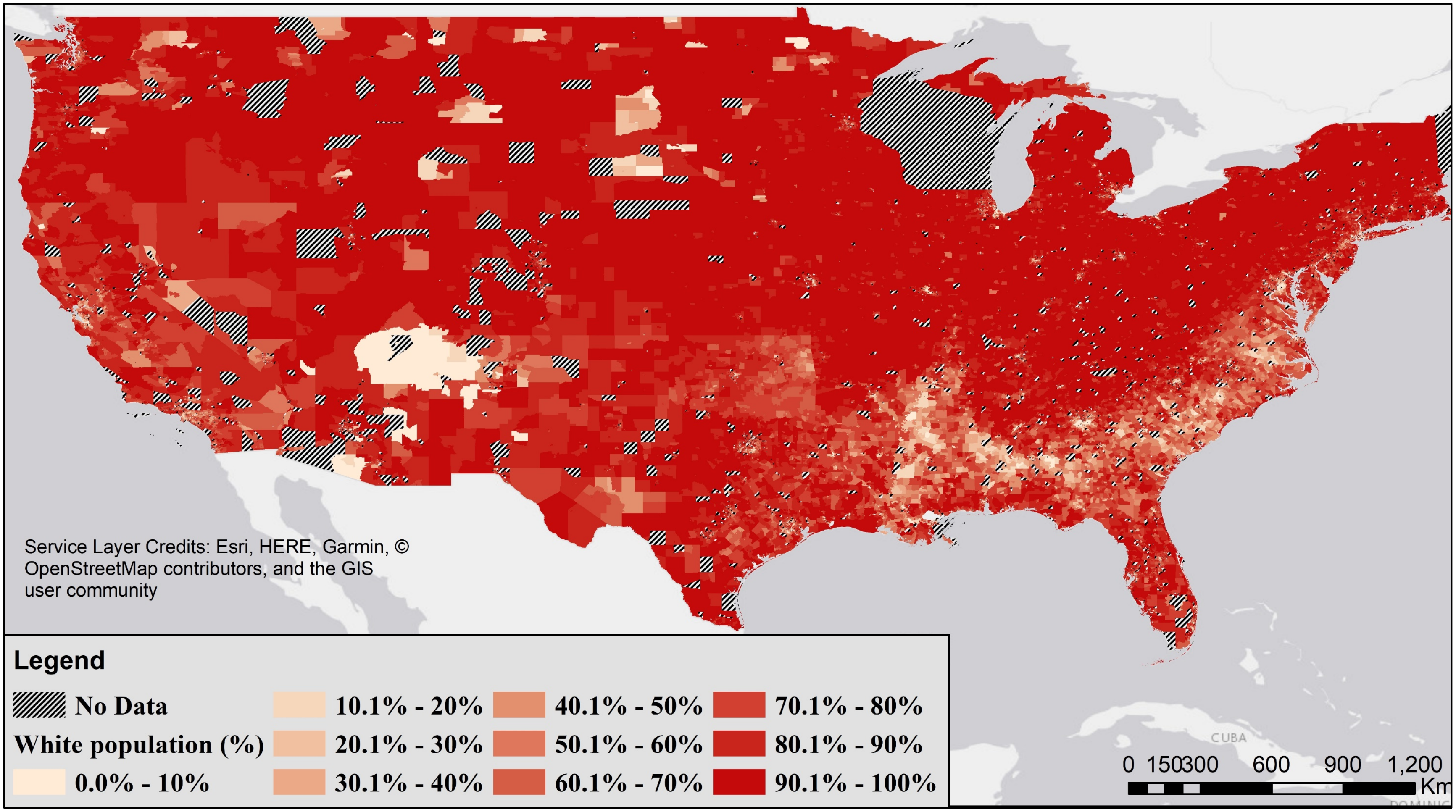

Figure 3. Percent white (variable name: white) in all census tracts with available data

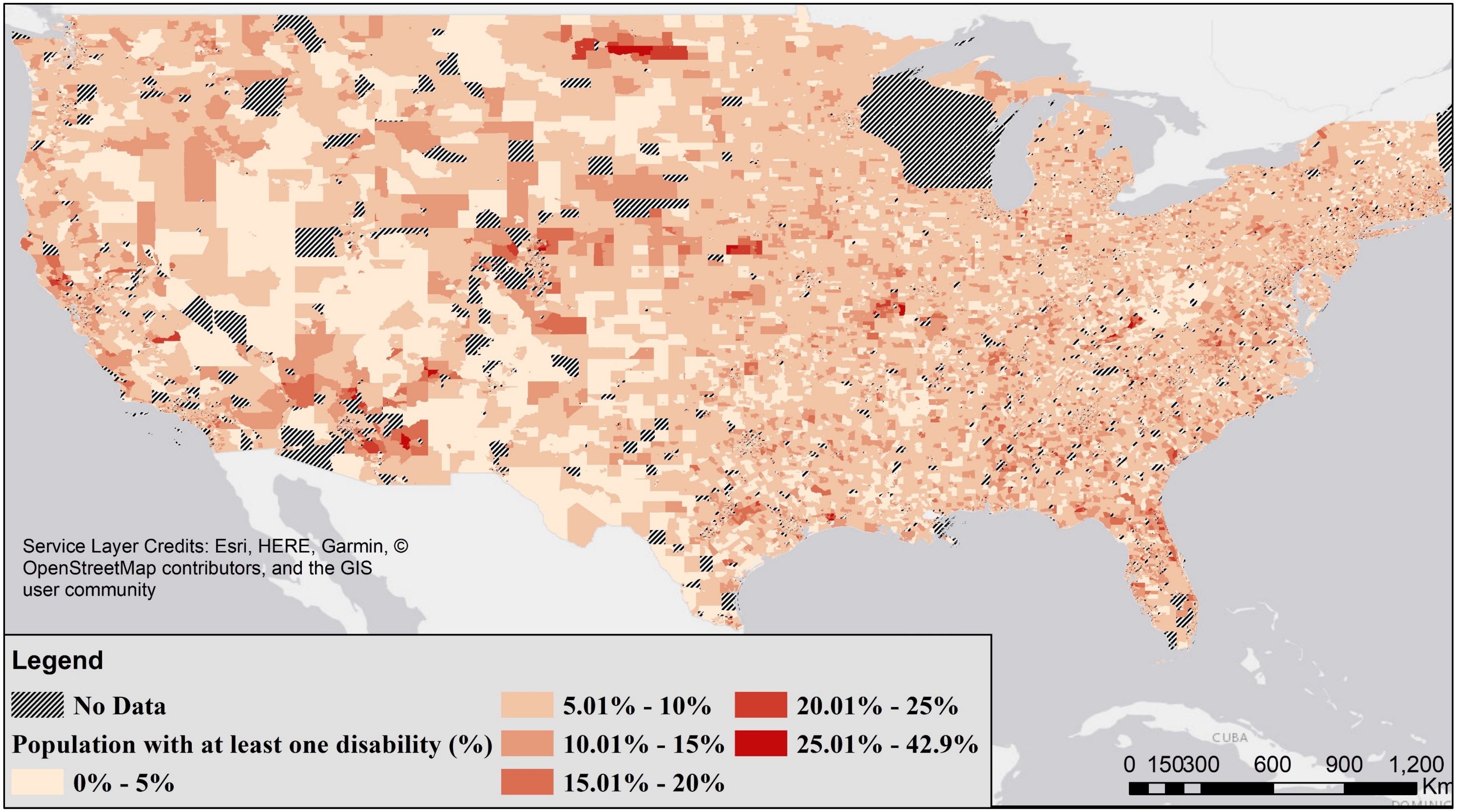

Figure 4. Percent of the population with at least one disability (variable name: disability) in all census tracts with available data

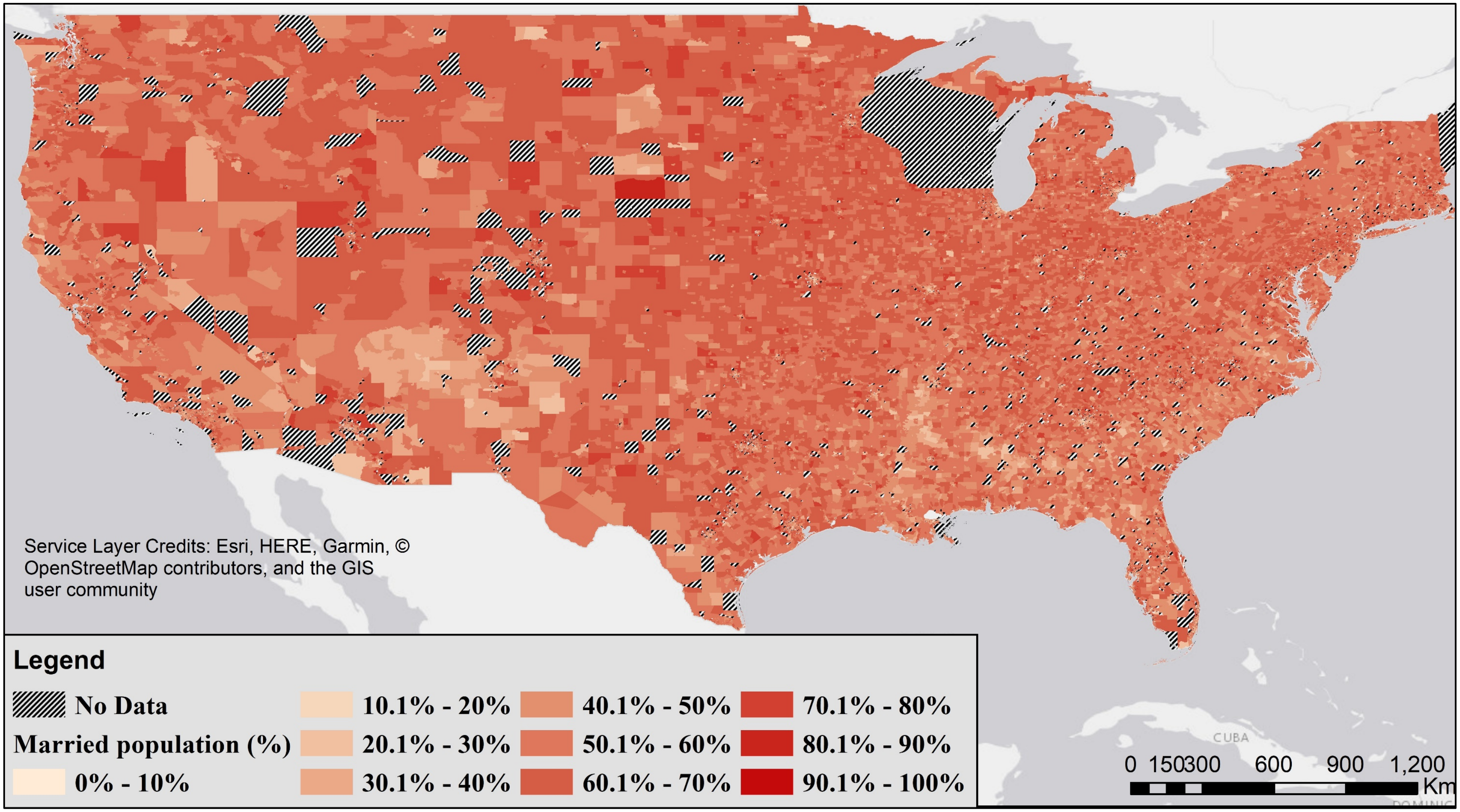

Figure 5. Percent of married people (variable name: married) in all census tracts with available data

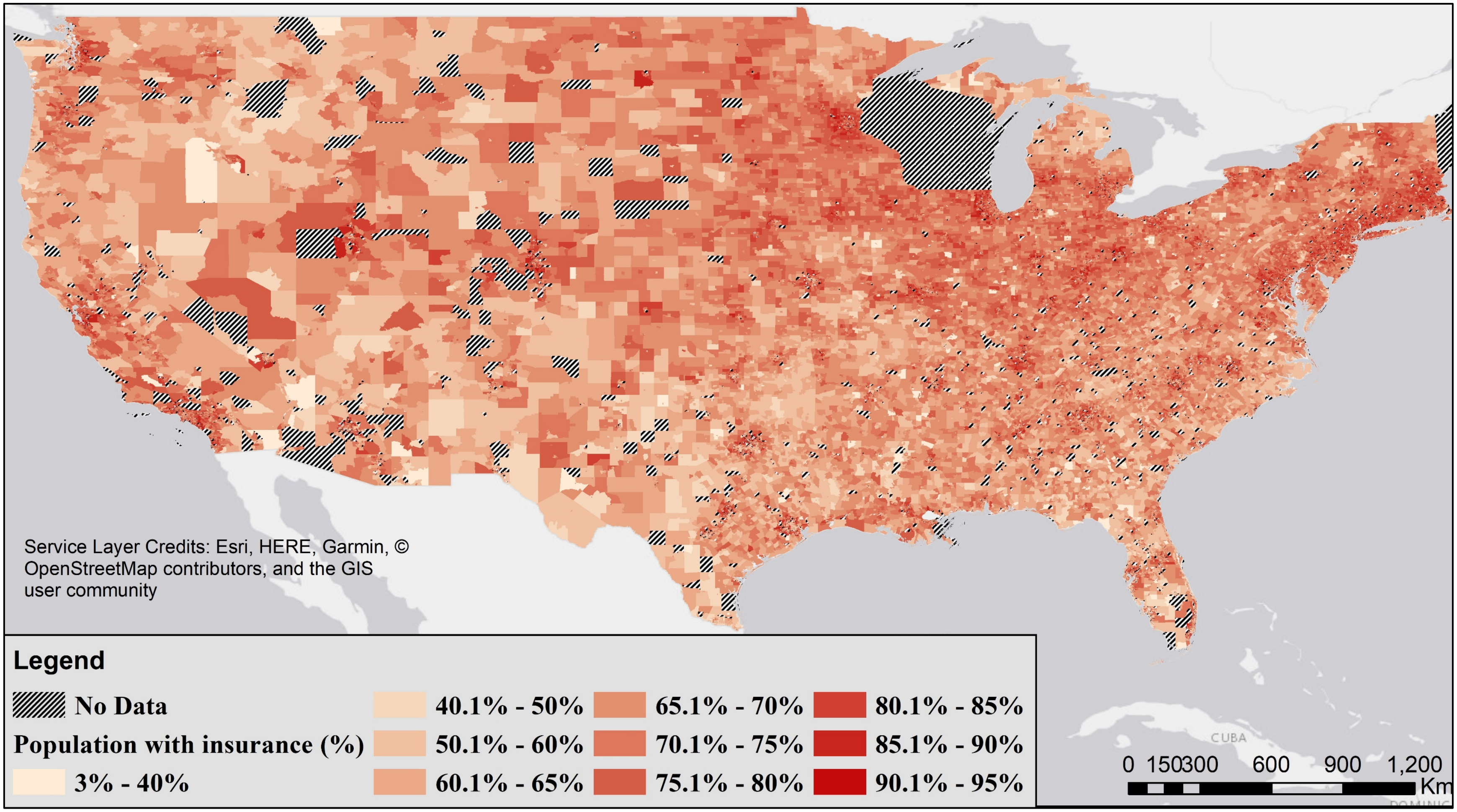

Figure 6. Percent of the population with at least one health insurance plan (variable name: insurance) in all census tracts with available data

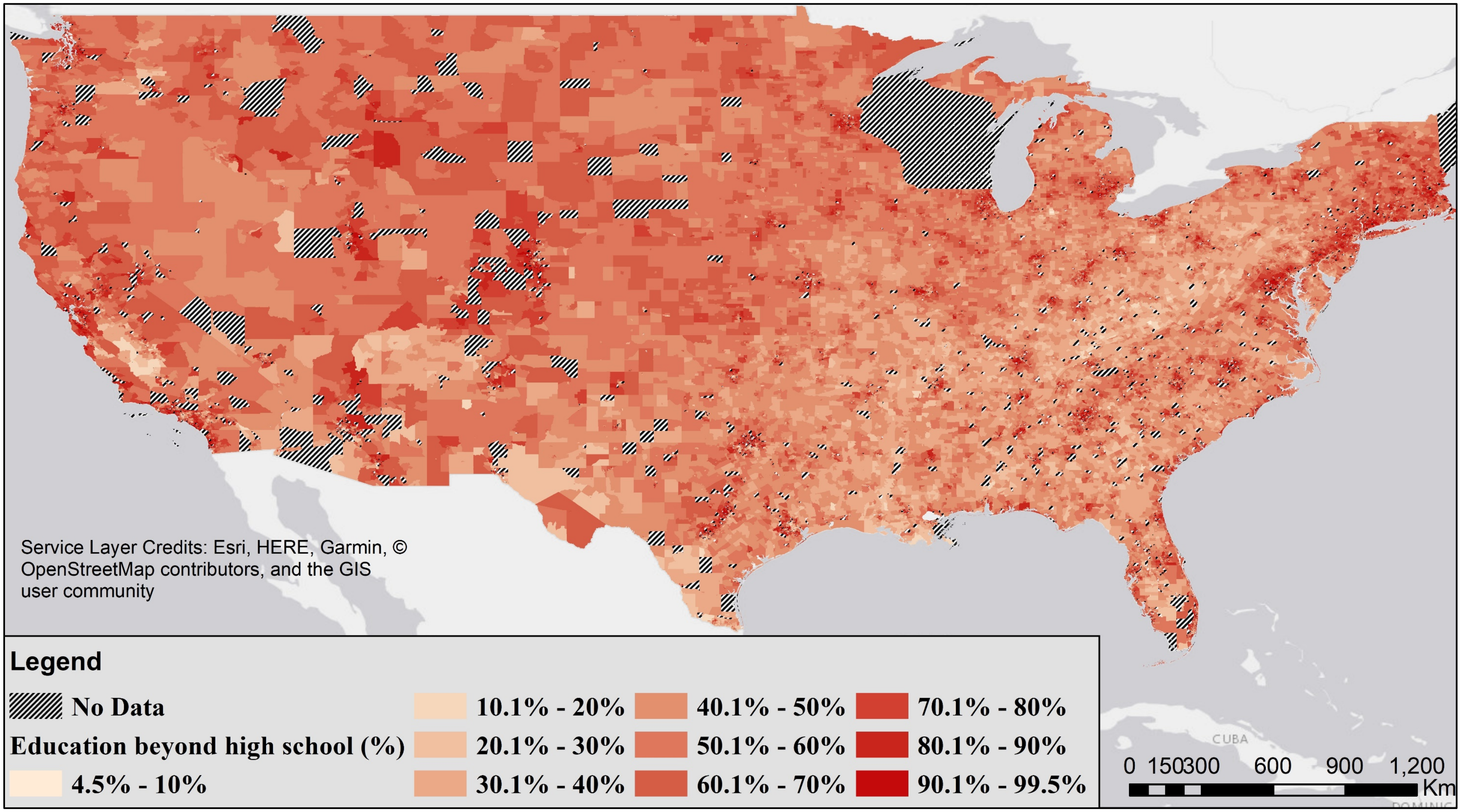

Figure 7. Percent of the population with education beyond a high school diploma (variable name: education) in all census tracts with available data

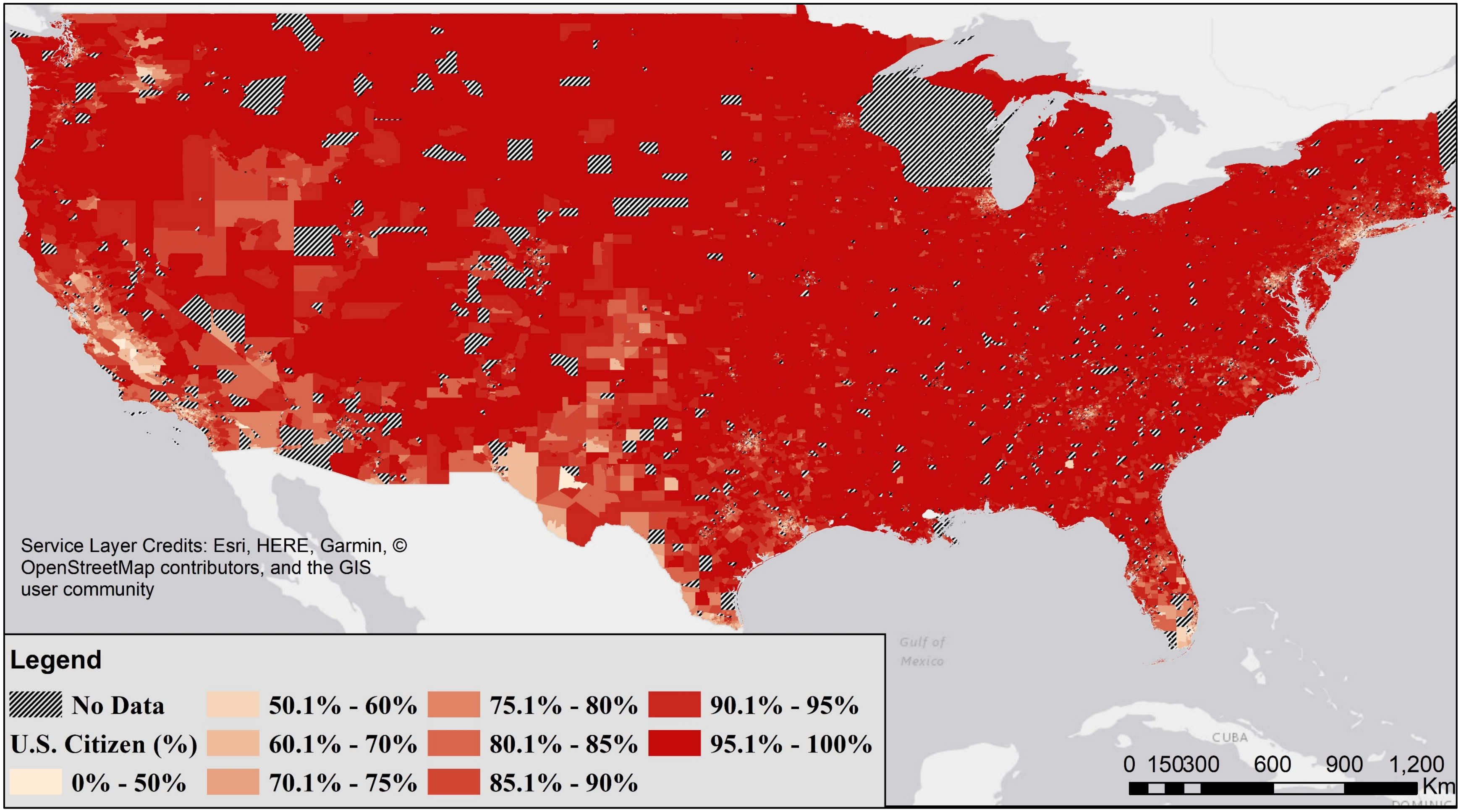

Figure 8. Percent U.S. citizens in each tract (variable name: citizenship) in all census tracts with available data

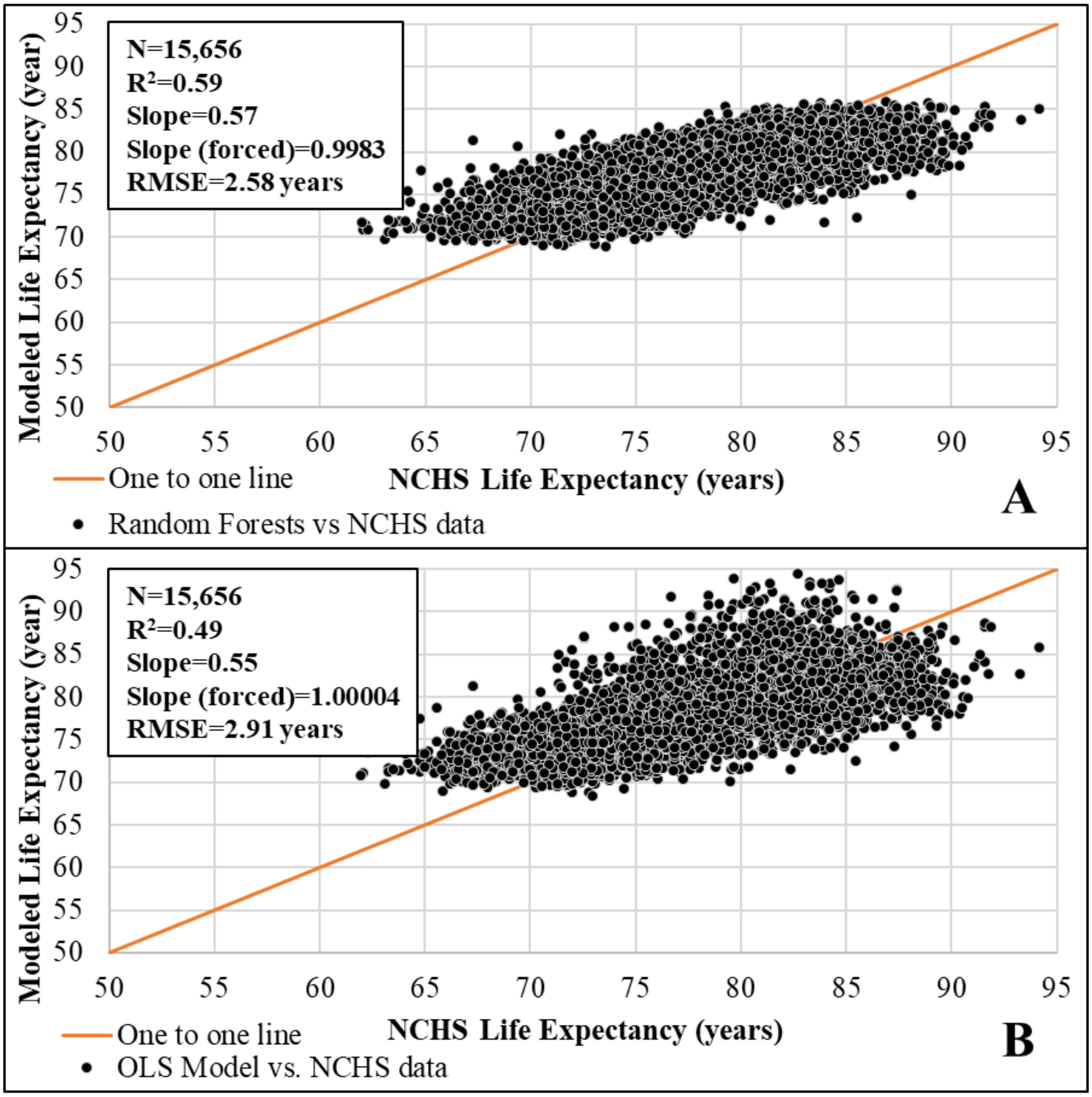

Figure 9. Performance of developed models in predicting life expectancy (LE) plotted against LEs estimated by the National Center for Health Statistics (NCHS). The validation dataset with 15,656 points were used to generate the plots. A) Random Forests model, B) OLS regression model

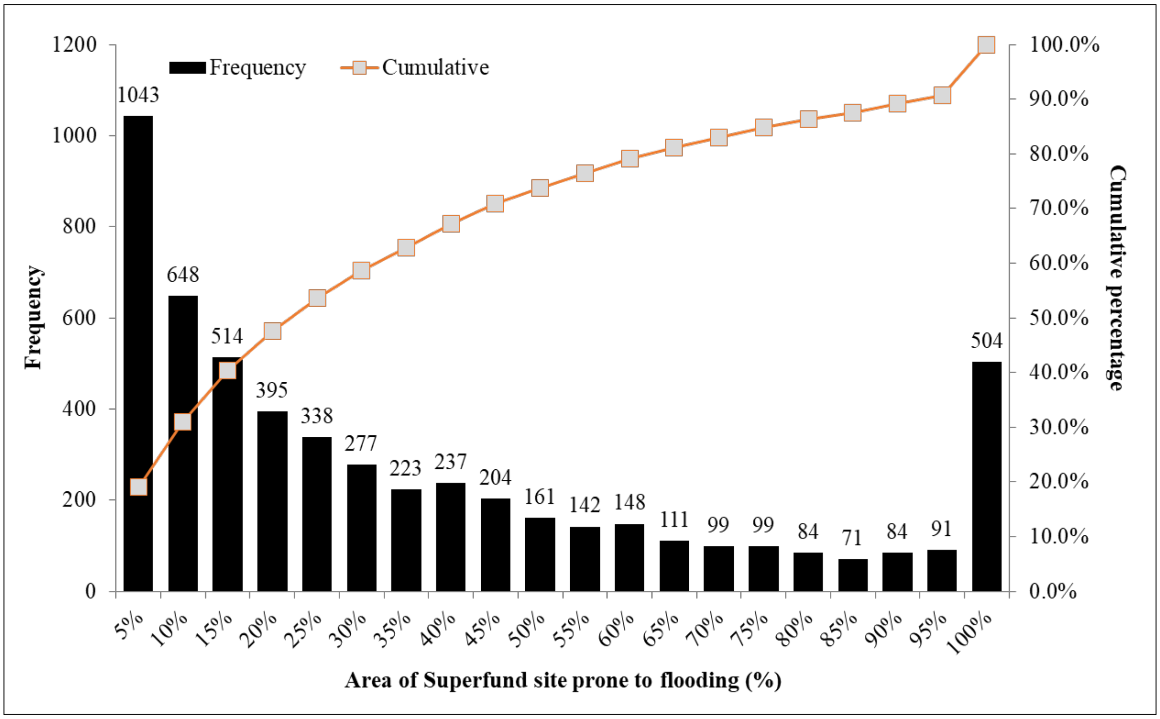

Figure 10. Frequency of Superfund sites for different ratios of flooding (areas located in floodplains defined by FEMA divided by total area)
