# Supplementary material for: The presence of Superfund sites as a determinant of life expectancy in the United States
Source: Nat Commun. 2021 Apr 13;12:1947. doi: 10.1038/s41467-021-22249-2 (PMC8044172; doi:10.1038/s41467-021-22249-2)
Supplement: Supplementary file 2 — Reporting Summary [file 41467_2021_22249_MOESM2_ESM.pdf]

nature research

Corresponding author(s): BDPR-NCOMMS-20-13472:

Last updated by author(s): Jan 27, 2021

Reporting Summary

Nature Research wishes to improve the reproducibility of the work that we publish. This form provides structure for consistency and transparency in reporting. For further information on Nature Research policies, see Authors & Referees and the Editorial Policy Checklist.

Statistics

For all statistical analyses, confirm that the following items are present in the figure legend, table legend, main text, or Methods section.

n/a	Confirmed	
☐	☒	The exact sample size (n) for each experimental group/condition, given as a discrete number and unit of measurement
☐	☒	A statement on whether measurements were taken from distinct samples or whether the same sample was measured repeatedly
☐	☒	The statistical test(s) used AND whether they are one- or two-sided<br>*Only common tests should be described solely by name; describe more complex techniques in the Methods section.*
☐	☒	A description of all covariates tested
☐	☒	A description of any assumptions or corrections, such as tests of normality and adjustment for multiple comparisons
☐	☒	A full description of the statistical parameters including central tendency (e.g. means) or other basic estimates (e.g. regression coefficient) AND variation (e.g. standard deviation) or associated estimates of uncertainty (e.g. confidence intervals)
☐	☒	For null hypothesis testing, the test statistic (e.g. F, t, r) with confidence intervals, effect sizes, degrees of freedom and P value noted<br>*Give P values as exact values whenever suitable.*
☒	☐	For Bayesian analysis, information on the choice of priors and Markov chain Monte Carlo settings
☐	☒	For hierarchical and complex designs, identification of the appropriate level for tests and full reporting of outcomes
☐	☒	Estimates of effect sizes (e.g. Cohen's d, Pearson's r), indicating how they were calculated

Our web collection on statistics for biologists contains articles on many of the points above.

Software and code

Policy information about availability of computer code

Data collection	A batch geocoding was performed based on the available addresses using the Geocodio website (https://www.geocod.io/)
Data analysis	Microsoft Excel 2016<br>IBM SPSS V26<br>MATLAB R2018a<br>ESRI ArcMap 10.6

For manuscripts utilizing custom algorithms or software that are central to the research but not yet described in published literature, software must be made available to editors/reviewers. We strongly encourage code deposition in a community repository (e.g. GitHub). See the Nature Research guidelines for submitting code & software for further information.

Data

Policy information about availability of data

All manuscripts must include a data availability statement. This statement should provide the following information, where applicable:
- Accession codes, unique identifiers, or web links for publicly available datasets
- A list of figures that have associated raw data
- A description of any restrictions on data availability

1) Sociodemographic data was downloaded from the National Historical Geographic Information System (NHGI) database (http://doi.org/10.18128/D050.V14.0)
2) Life expectancy data were downloaded from the National Center for Health Statistics (NCHS) database (https://www.cdc.gov/nchs/nvss/usaleep/usaleep.html). For two states, Maine and Wisconsin, the LE data were not available in the database due to lack of geocoded death records
3) All available information on active and archived Superfund sites was downloaded from the EPA's Superfund Enterprise Management System database (https://cumulis.epa.gov/supercpad/cursites/srchsites.cfm)
4) Flood hazard map data were downloaded from the National Flood Hazard Layer (NFHL) database maintained by the Federal Emergency Management Agency (FEMA) (https://www.floodmaps.fema.gov/NFHL/status.shtml.)

Figure 1 has raw data from source 1 and 3 (census tract shapefile, and Superfund NPL status), Figure 2a has raw data from source 2, and Supplementary Figures 1-8 in the Supplementary Information (SI) has raw data from source 1. All generated results (including the raw data) in both tabulated and shapefile formats have been deposited in the Open Science Framework (OSF) under the name ""KiaghadiEtAl_Nature_Communications_DATA"" and are accessible through Kiaghadi, A. The Presence of Superfund Sites as a Determinant of Life Expectancy in the United States, KiaghadiEtAl_Nature_Communications_Data, https://www.doi.org/10.17605/OSF.IO/EX2ZK (2021).

Field-specific reporting

Please select the one below that is the best fit for your research. If you are not sure, read the appropriate sections before making your selection.

☐ Life sciences ☐ Behavioural & social sciences ☒ Ecological, evolutionary & environmental sciences

For a reference copy of the document with all sections, see nature.com/documents/nr-reporting-summary-flat.pdf

Ecological, evolutionary & environmental sciences study design

All studies must disclose on these points even when the disclosure is negative.

Study description	In this study, we assess to what extent the presence of hazardous waste and Superfund sites could alter the Life Expectancy (LE) independently and in the context of other sociodemographic determinants. A nationwide geocoded statistical modeling analysis at the census tract level was undertaken to demonstrate and estimate the potential of impact and the magnitude of such impact.
Research sample	In this study we used existing databases at the census tract level for the entire contiguous USA. The list of data we used included:<br><br>Census variables: population above 60 years old, median income and income per capita in U.S. dollars, population below the poverty line, race, population with at least one disability, marriage status, population with at least one health insurance plan, population with education beyond high school diploma, U.S. citizenship for the entire USA<br><br>Flooding variables: Areas located in 100-yr and 500-yr, floodway, areas with minimum flood hazard, areas with reduced flood risk due to levees, coastal flooding. These variables were spatially joined to the census tracts using ArcMap.<br><br>EPA Superfund data: Locations, National Priority List status, clean-up status. These variables were spatially joined to the census tracts using ArcMap.<br><br>Life Expectancy estimated data by the National Center for Health Statistics (NCHS) at census tract level.
Sampling strategy	All available data were used.
Data collection	In this study, we used existing databases. All data were manually downloaded from the Online databases using personal computers. The authors and undergraduate researchers working in the research labs were in charge of downloading the data.
Timing and spatial scale	All data were continuously downloaded within a month.
Data exclusions	In all of the analyses, we excluded census tracts in two states, Maine and Wisconsin, because the Life Expectancy data were not available in the database due to lack of geocoded death records.
Reproducibility	not applicable to this study
Randomization	not applicable to this study
Blinding	not applicable to this study

Did the study involve field work? ☐ Yes ☒ No

Reporting for specific materials, systems and methods

We require information from authors about some types of materials, experimental systems and methods used in many studies. Here, indicate whether each material, system or method listed is relevant to your study. If you are not sure if a list item applies to your research, read the appropriate section before selecting a response.

Materials & experimental systems

n/a	Involved in the study
☒	☐ Antibodies
☒	☐ Eukaryotic cell lines
☒	☐ Palaeontology
☒	☐ Animals and other organisms
☒	☐ Human research participants
☒	☐ Clinical data

Methods

n/a	Involved in the study
☒	☐ ChIP-seq
☒	☐ Flow cytometry
☒	☐ MRI-based neuroimaging
